# Dynamic prediction in functional concurrent regression with an application to child growth

**DOI:** 10.1002/sim.7582

**Published:** 2017-12-11

**Authors:** Andrew Leroux, Luo Xiao, Ciprian Crainiceanu, William Checkley

**Affiliations:** ^1^ Department of Biostatistics Johns Hopkins University Baltimore MD 21205 USA; ^2^ Department of Statistics North Carolina State University Raleigh NC 27606 USA; ^3^ School of Medicine Johns Hopkins University Baltimore MD 21205 USA

**Keywords:** covariance function, face, fPCA, longitudinal data, mixed effects, penalized splines, sparse functional data

## Abstract

In many studies, it is of interest to predict the future trajectory of subjects based on their historical data, referred to as dynamic prediction. Mixed effects models have traditionally been used for dynamic prediction. However, the commonly used random intercept and slope model is often not sufficiently flexible for modeling subject‐specific trajectories. In addition, there may be useful exposures/predictors of interest that are measured concurrently with the outcome, complicating dynamic prediction. To address these problems, we propose a dynamic functional concurrent regression model to handle the case where both the functional response and the functional predictors are irregularly measured. Currently, such a model cannot be fit by existing software. We apply the model to dynamically predict children's length conditional on prior length, weight, and baseline covariates. Inference on model parameters and subject‐specific trajectories is conducted using the mixed effects representation of the proposed model. An extensive simulation study shows that the dynamic functional regression model provides more accurate estimation and inference than existing methods. Methods are supported by fast, flexible, open source software that uses heavily tested smoothing techniques.

## 1 INTRODUCTION

In many biological and epidemiological studies, sampling is conducted at multiple time points resulting in longitudinal data that exhibit within‐subject correlation. Traditionally, longitudinal data have been analyzed using either marginal models^1^ or conditional mixed effect models.(2, 3, 4) Both approaches are parametric and are not designed to account for subtle or strong departures from the assumed parametric trends. This problem can manifest in a number of ways in longitudinal data, including autocorrelation in the residuals of random intercept/slope models.^5^ To address such challenges, one may consider using methods for functional data, which allow more flexible modeling of subject‐specific random curves. A random functional intercept model can be understood as a special case of a broader class of models referred to as functional mixed effects models. However, functional mixed models are complex, and the computational burden for fitting them is nontrivial.

A computationally feasible approach to estimating functional mixed effects models is to combine semiparametric regression techniques^6^ with functional data analysis.^7^ Arguably, the functional mixed effects framework was first introduced by Guo^8^ and was further developed in subsequent years.(9, 10, 11, 12, 13, 14, 15, 16, 17, 18, 19) Recently, Scheipl et al^20^ provided a general framework for fitting functional mixed effects models based on the idea of incorporating nonparametric smoothing approaches into the standard mixed effects modeling framework.^6^

Although the aforementioned advances in methodology are substantial, the release of accompanying software has been limited. As a result, use of functional mixed effects models in scientific studies has lagged behind traditional methods in spite of the wide range of data types amenable to this modeling framework. To the best of our knowledge, only the R package *refund* 21 has provided functions that fit standard functional mixed effects models. Despite the varied models that can be fit using the *refund* package, the estimation of a functional concurrent regression (FCR) model with both irregularly measured response and predictors is not currently possible. Moreover, neither *refund* nor the standard *R* packages for fitting mixed effects models, *lme4* 22 and *nlme*,^23^ are able to readily handle dynamic prediction. Specifically, the current software packages do not allow for predictions on subjects not included in the model fitting procedure. With reproducibility and dissemination of our methods as a primary goal, we propose a dynamic FCR model applied to growth curve data in children and provide an accompanying *R* package, *fcr*, capable of fitting this class of models.

Dynamic prediction in functional data analysis is relatively new. Recently, several authors have proposed methods, which allow for prediction of partially observed functional data via functional principal component analysis.(24, 25, 26, 27, 28) However, these works involve only functional responses and do not consider subject‐specific predictors of any form. The proposed work fills this gap using a functional concurrent model. Indeed, in our application, subject predictions are based on time‐invariant covariates (sex), time‐varying covariates (weight), and past length measurements. The effects of both time‐varying and time‐invariant covariates are modeled using time‐varying coefficients.^29^ A child's past length measurements and their length at a future age are modeled by a child‐specific functional random intercept. While a random intercept/random slope model could be used to characterize child‐specific growth trajectories of height, such an approach is not well suited for the nonlinear growth trajectories of children (see Figure 2).

The rest of the paper is organized as follows. In Section 2, we describe the motivating data and provide an exploratory analysis. In Sections 3 and 4, we introduce the dynamic FCR model and propose an estimation method. In Section 5, we apply the method to the CONTENT growth data and discuss our findings. In Section 6, we conduct a simulation study to evaluate the performance of the proposed functional mixed effects model. We conclude the paper with a discussion in Section 7.

## 2 THE CONTENT CHILD GROWTH DATA

The CONTENT study was conducted in 2 peri‐urban shanty towns approximately 25 km south of central Lima in Peru. The goal of the study was to assess the impact of a particular bacterial infection, *Helicobacter pylori*, on child growth.^30^ To that end, anthropometric measurements on 215 children (106 boys and 109 girls) were collected periodically starting from birth. We focus on data collected at 547 unique time points between 0 and 24 months. Children have an average of 39 anthropometric measurements each, with measurements observed more frequently during the first few months of life.

We examine 3 World Health Organization (WHO)‐defined anthropometric measurements: length‐for‐age *z*‐score (LAZ), weight‐for‐length *z*‐score, and weight‐for‐age *z*‐score (WAZ).^31^ The *z*‐scores are calculated using the age‐ and sex‐specific WHO standard references. This *z*‐scoring procedure is standard in growth curve modeling and provides data on a scale relative to a standard of growth, with a *z*‐score of 0 representing the WHO‐defined average for that age and sex. *Z*‐scores greater (smaller) than 0 indicate above (below) average measurement relative to the WHO standard in that category. As a result, the *z*‐scores can be compared within‐ and between‐children over time. For example, a *z*‐score of 0 at 3 months compared with a *z*‐score of 1 at 6 months for the same child indicates that the child is at the WHO average length at 3 months, but above the WHO average by 6 months.

**Figure 1:**
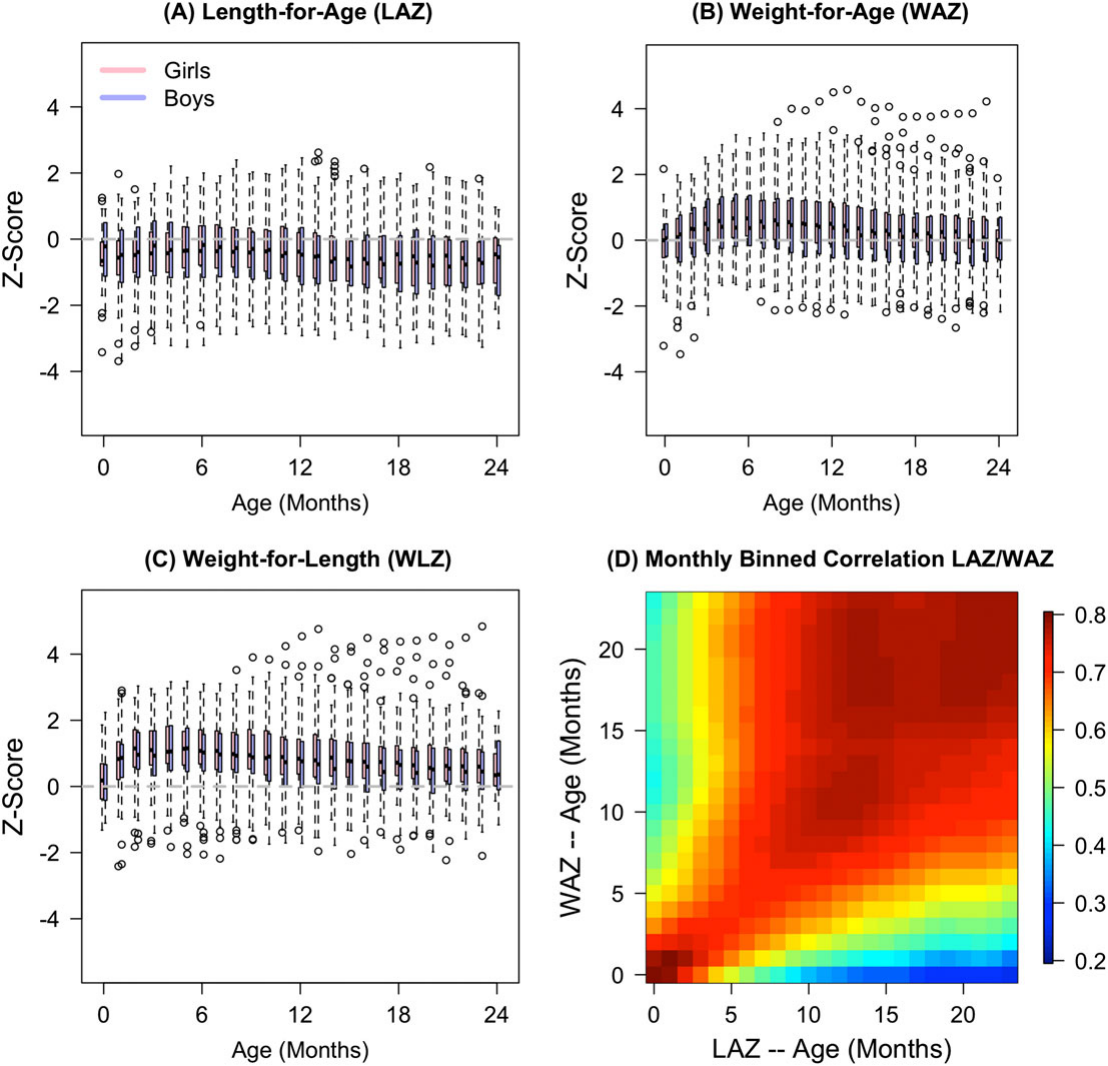
Distribution of, A, length‐for‐age z‐scores (LAZ), B, weight‐for‐age z‐scores (WAZ), C, weight‐for‐length z‐scores (WLZ) binned into monthly categories, and, D, estimated empirical correlation between LAZ and WAZ at each month. Correlations were calculated using Z‐scores projected onto an evenly spaced grid of ages {0.5,1.5,…,23.5} for all subjects using face::face.sparse() applied separately to LAZ and WAZ

Figure 1A‐C displays the marginal distributions of anthropometric measurements binned into monthly intervals. Several features of the data can be observed. First, these children are, on average, below the WHO‐standard average length and above the WHO‐standard average weight. These growth characteristics have been reported in similar populations.(32, 33) In addition, the trend in *z*‐scores for boys and girls is similar across all ages. Furthermore, there appears to be a disproportionate weight gain relative to length gain for the 0‐ to 3‐month period (see Figure 1C).

Figure 1D displays the estimated correlation between subjects' LAZ(*t*) and WAZ(*s*) for all months *t*,*s*. Correlations are generally high between measurements taken close in time and are higher closer to birth (notice the shades of red along the main diagonal and the darker red shades in the lower left corner). Correlations remain high even at larger time lags (months 20+ ) with values above 0.4 for many time pairs.

**Figure 2:**
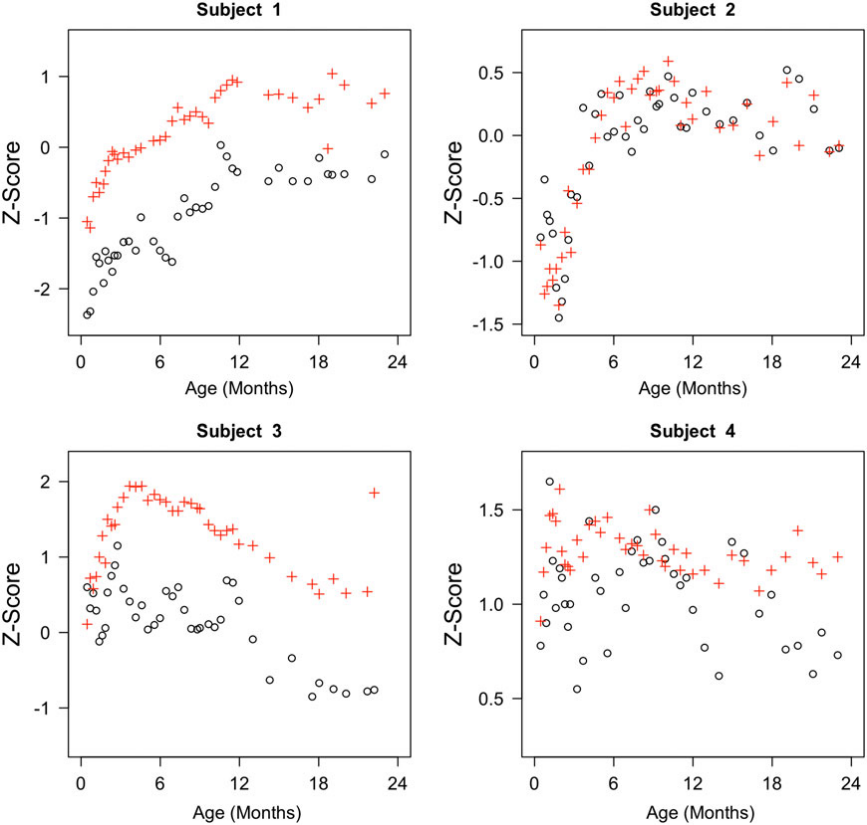
Length‐for‐age z‐score and weight‐for‐age z‐score curves for 4 children. Length‐for‐age z‐score is presented as ∘ and weight‐for‐age z‐score is presented as +

Unlike nonstandardized length (height) and weight measurements, which increase continuously for most children, the trend in *z*‐scores is quite different. Figure 2 displays the LAZ and WAZ scores for 4 children, indicating strong nonlinear characteristics of the curves. These 4 subjects are representative of subjects' diverse growth patterns. Subject 1 has a fairly steady increasing trajectory up to 12 months followed by a period of relatively stable growth from 12 to 24 months. In contrast, subjects 2 and 3 experience steep declines (increases) followed by steep increases (declines) in early life. Following a period of relatively consistent growth, near 12 months, subject 3 undergoes a period of rapid decline in standardized length, suggesting some degree of stunting. Accounting for the diverse and markedly nonlinear growth patterns observed requires flexible models. Regarding the relationship between LAZ and WAZ, Figure 2 suggests these 2 *z*‐scores exhibit positive correlations, reinforcing the observed population level correlations displayed in Figure 1.

**Figure 3:**
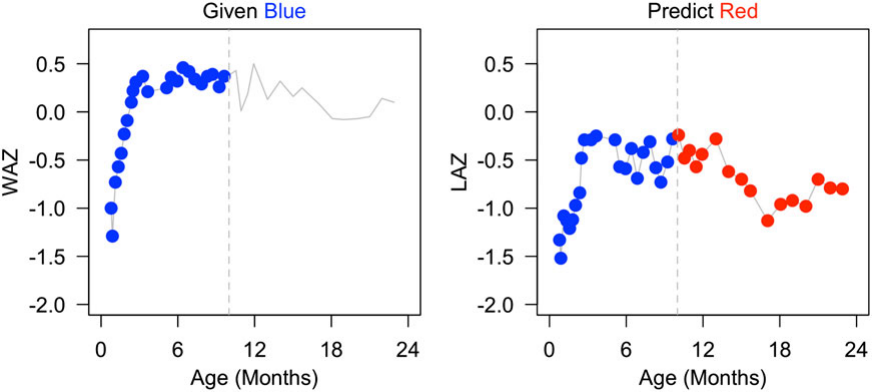
Illustration of dynamic prediction using a subject from the CONTENT study. Conditional on the weight‐for‐age z‐score (WAZ) and length‐for‐age z‐score (LAZ) data in blue, the interest is to predict the length‐for‐age z‐score (LAZ) in red

Since it is known that delayed growth development is associated with a number of negative health outcomes, it is of interest to identify children at risk of delayed growth and/or growth stunting as early as possible. Such identification via growth trajectories can only be achieved using a dynamic approach. Indeed, a post hoc analysis can only identify that certain children were stunted and cannot inform the development of potential interventions. Correspondingly, the primary goal of our application is to build a model that can dynamically predict length‐for‐age *z*‐scores in the first 2 years of life. As there is a clear association between weight and length in children, we aim to build a model that addresses this relationship that is compatible with dynamic prediction. The procedure requires the use of historical information available up to a particular time point to predict the future trajectory. In Figure 3, we provide an illustration of the dynamic prediction of LAZ based on observed LAZ and WAZ. Finally, we propose an inferential framework that provides confidence intervals both for curve predictions and model parameters.

## 3 MODEL

### 3.1 Notation

Let *t* denote age in months. For subject *i*, let *Y*_*i*_(*t*) be the LAZ at age *t*, *Z*_*i*_(*t*) be the WAZ at age *t*, and *X*_*i*_ be the sex indicator (1 for boys and 0 for girls). The observed data are [{*t*_*ij*_,*Y*_*ij*_=*Y*_*i*_(*t*_*ij*_),*Z*_*ij*_=*Z*_*i*_(*t*_*ij*_),*X*_*i*_}, 1≤*j*≤*m*_*i*_,1≤*i*≤*N*], where *t*_*ij*_ denotes the *j*th visit time for subject *i*, *m*_*i*_ is the number of observations for subject *i*, and *N* is the total number of subjects.

### 3.2 Model

We consider the following model:

(1) $$Y_{i}(t)=f_{0}(t)+X_{i}\;f_{1}(t)+Z_{i}(t)\;f_{2}(t)+b_{i}(t)+\epsilon_{i}(t),$$

where *f*_0_(·) is the population intercept function, *f*_1_(·) models the sex difference, *f*_2_(·) models the concurrent association of WAZ with LAZ, *b*_*i*_(·) models the subject‐specific random functional deviation of subject *i* from *f*_0_(·), and *ϵ*_*i*_(*t*) are independent random errors. More specifically, we model *b*_*i*_(*t*) as a zero‐mean Gaussian process with a covariance function cov{*b*_*i*_(*s*),*b*_*i*_(*t*)}=*C*(*s*,*t*) and assume that *ϵ*_*i*_(*t*) are independent and identically distributed as $N(0,\sigma_{\epsilon }^{2})$. We assume that *b*_*i*_(·) and *ϵ*_*i*_(*t*_*ij*_) are mutually independent across subjects.

The WAZ process is also measured with error, and we assume that for subject *i*, the observed WAZ is $Z_{i,\text{obs}}(t_{ij})=Z_{i}(t_{ij})+\epsilon_{i}^{z}(t_{ij})$. Here, $\epsilon_{i}^{z}(t_{ij})$ is the term for measurement errors and is assumed to be independent and identically distributed as $N(0,\sigma_{\epsilon z}^{2})$. We let *Z*_*i*_(*t*_*ij*_)=*μ*_*z*_(*t*_*ij*_)+*b*_*iz*_(*t*_*ij*_), where *μ*_*z*_(·) is the population mean of WAZ and *b*_*iz*_(·) is the subject‐specific random functional deviation from the population mean. We model *b*_*iz*_(·) as a stochastic process with zero mean and a covariance function cov{*b*_*iz*_(*s*),*b*_*iz*_(*t*)}=*C*_*z*_(*s*,*t*) and assume that *b*_*iz*_(·) are independent across subjects. It is possible to use additional predictors in estimating *Z*_*i*_(*t*_*ij*_), although this option is not explored here.

Model (1) is a concurrent effect model for the time‐varying covariate *Z*_*i*_(*t*_*ij*_). We show how this concurrent modeling framework can be used for dynamic prediction in Section 4. The historical information is incorporated implicitly in model (1) via the functional random effect *b*_*i*_(*t*). We have found this implicit approach to provide an excellent alternative to the explicit modeling of historical functional data. Thus, our proposal will add to the literature and will provide alternative inferential approaches that are especially useful in the case of sparse longitudinal data.

The explicit modeling of historical functional data was introduced by Malfait and Ramsay,^34^ while Şentürk and Müller^35^ allowed the response to depend on smooth functions of arbitrarily many lagged covariate values. Building on the idea of historical models, Şentürk and Müller^36^ used the concept of a “*history index*” to allow for a response to depend only on the recent historical values of a covariate. Given the existence of these models, it is tempting to consider a full historical effect term $\int_{0}^{t}\beta (s,t)Z_{i}(s)ds$, where *β*(*s*,*t*) is an unknown bivariate function. We do not adopt such a specification because it did not improve fit to the CONTENT data.

### 3.3 Model estimation

One of our main contributions is to provide explicit methods and formulas to fit model (1). This is not trivial, as such models have not been implemented before, and they require some heavy notation and attention to detail. First, we conduct a functional principal component analysis on the *Z*_*i*,obs_(*t*_*ij*_)s using the R package *face*. This results in estimates ${\hat{\mu }}_{z}(t)$, ${Ĉ}_{z}(s,t)$, and ${\hat{\sigma }}_{\epsilon z}^{2}$. Then, conditional on the observed data $Z_{i,\text{obs}}={\left\{Z_{i,\text{obs}}(t_{i1}),\text{⋯},Z_{i,\text{obs}}(t_{im_{i}})\right\}}^{\prime }$, we predict *Z*_*i*_(*t*) by its best linear unbiased predictor (BLUP), ${\tilde{Z}}_{i}(t)={\hat{\mu }}_{z}(t)+{Ĉ}_{z}{\left(t,t_{i}\right)}^{\prime }{\left\{{Ĉ}_{z}(t_{i},t_{i})+{\hat{\sigma }}_{\epsilon z}^{2}I_{m_{i}}\right\}}^{-1}\{Z_{i,\text{obs}}-{\hat{\mu }}_{z}(t_{i})\}$,^37^ where $t_{i}={\left(t_{i1},\text{⋯},t_{im_{i}}\right)}^{\prime }\in R^{m_{i}}$, ${Ĉ}_{z}(t,t_{i})={\left\{C_{z}(t,t_{i1}),\text{⋯},C_{z}(t,t_{im_{i}})\right\}}^{\prime }\in R^{m_{i}}$, ${\hat{\mu }}_{z}(t_{i})={\left\{\mu_{z}(t_{i1}),\text{⋯},\mu_{z}(t_{im_{i}})\right\}}^{\prime }\in R^{m_{i}}$, and ${Ĉ}_{z}(t_{i},t_{i})={\left\{{Ĉ}_{z}(t_{ij},t_{ik})\right\}}_{1\leq j,k\leq m_{i}}\in R^{m_{i}\times m_{i}}$. We then substitute ${\tilde{Z}}_{i}(t_{ij})$ for *Z*_*i*_(*t*_*ij*_) in model (1).

For notational convenience, we rewrite model (1) as

(2) $$Y_{ij}=f_{0}(t_{ij})+\sum_{p=1}^{P}z_{p,ij}\;f_{p}(t_{ij})+b_{i}(t_{ij})+\epsilon_{ij},$$

where *P*=2, *z*_1,*ij*_=*X*_*i*_, *z*_2,*ij*_=*Z*_*ij*_. Models for functional responses have been studied in Ivanescu et al^38^ and Scheipl et al^20^ and are implemented via the *pffr* function in the R package *refund*. However, *pffr* currently accommodates only time‐varying predictors, which are measured on a regular grid. As a result, *pffr* cannot be used to implement the method described above using the CONTENT data. Below we detail our estimation method using *P*‐splines,^39^ and we provide an R package that extends the *pffr* function for concurrent models with irregularly measured time‐varying covariates.

We approximate *f*_*p*_(·) by a linear combination of B‐spline basis functions. More specifically, let $B(t)={\left\{B_{1}(t),\text{⋯},B_{c}(t)\right\}}^{\prime }$ be a sequence of B‐spline basis functions evaluated at *t*, where *c* is the number of interior knots plus the order of the B‐spline basis functions. We use cubic B‐splines and place interior knots at the quantiles of the observed time points.^40^ For simplicity, we use the same basis functions for all *f*_*p*_,*p*=0,1,…,*P*. Then we let $f_{p}(t)=B{\left(t\right)}^{\prime }\theta_{p}$, where $\theta_{p}={\left(\theta_{p1},\text{⋯},\theta_{pc}\right)}^{\prime }$ is the vector of coefficients.

We also approximate the random subject‐specific curves *b*_*i*_(*t*) using B‐spline basis functions with $b_{i}(t)=\sum_{k=1}^{c}u_{ik}B_{k}(t)$, where $u_{i}={\left(u_{i1},\text{⋯},u_{ic}\right)}^{\prime }\in R^{c}$. Unlike Chen and Wang^12^ who used a small number of basis functions, we use a relatively large number of basis functions to allow for increased modeling flexibility of subject‐specific functions. We assume that **u**_*i*_ follows a multivariate normal distribution with zero mean and covariance $\Gamma ={\left(\gamma_{k\ell }\right)}_{1\leq k,\ell \leq c}\in R^{c\times c}$. This implies that *C*(*s*,*t*)= ∑_1≤*k*,*ℓ*≤*c*_ *γ*_*kℓ*_ *B*_*k*_(*s*)*B*_*ℓ*_(*t*). The covariance matrix **Γ** is estimated from the data. Details are provided in Section 3.4.

For each *i*, we let $y_{i}={\left(Y_{i1},\text{⋯},Y_{im_{i}}\right)}^{\prime }\in R^{m_{i}}$, $z_{p,i}={\left(z_{p,i1},\text{⋯},z_{p,im_{i}}\right)}^{\prime }\in R^{m_{i}}$, $B_{i}={\left[B(t_{i1}),\text{⋯},B(t_{im_{i}})\right]}^{\prime }\in R^{m_{i}\times c}$, and $X_{i}=[B_{i},\text{diag}(z_{1,i})B_{i},\text{⋯},\text{diag}(z_{P,i})B_{i}]\in R^{m_{i}\times (P+1)c}$. With this notation, we have $E(y_{i})=X_{i}\theta$, where $\theta ={\left(\theta_{0},\text{⋯},\theta_{P}\right)}^{\prime }\in R^{(P+1)c}$, and $V_{i}=\text{Cov}(y_{i})=B_{i}\Gamma B_{i}^{\prime }+\sigma_{\epsilon }^{2}I_{m_{i}}$. The generalized least squares estimate for ***θ*** is then

$$\hat{\theta }=\arg\min_{\theta }N^{-1}\sum_{i=1}^{N}{\left(y_{i}-X_{i}\theta \right)}^{\prime }V_{i}^{-1}(y_{i}-X_{i}\theta ).$$

Because regression splines tend to over‐fit, we enforce smoothness in the estimated coefficient functions by imposing a penalty on ***θ*** as suggested by Eilers and Marx.^39^ Specifically, we let **P**=blockdiag(**P**_0_,**P**_**1**_,…,**P**_*P*_) and for $p\in \{0,1,\text{⋯},P\},P_{p}=\lambda_{p}D^{\prime }D$, where **D** is the (*c*−*o*)×*c* differencing matrix and *o* is the order of the difference penalty.^41^ We use *o*=2 throughout the paper. Thus, we estimate ***θ*** by penalized generalized least squares

$$\hat{\theta }=\arg\min_{\theta }N^{-1}\sum_{i=1}^{N}{\left(y_{i}-X_{i}\theta \right)}^{\prime }V_{i}^{-1}(y_{i}-X_{i}\theta )+\theta^{\prime }P\theta$$

and we obtain

(3) $$\hat{\theta }={\left(N^{-1}\sum_{i=1}^{N}X_{i}^{\prime }V_{i}^{-1}X_{i}+P\right)}^{-1}\left(N^{-1}\sum_{i=1}^{N}X_{i}^{\prime }V_{i}^{-1}y_{i}\right).$$

Let $\hat{\theta }={\left({\hat{\theta }}_{0},\text{⋯},{\hat{\theta }}_{P}\right)}^{\prime }$ such that ${\hat{\theta }}_{p}$ is the corresponding estimate of ***θ***_*p*_. Then each coefficient function can be estimated as ${\hat{f}}_{p}(t)=B{\left(t\right)}^{\prime }{\hat{\theta }}_{p}$.

Using simple algebra, it follows that

$$\text{Cov}(\hat{\theta })={\left(N^{-1}\sum_{i=1}^{N}X_{i}^{\prime }V_{i}^{-1}X_{i}+P\right)}^{-1}\left(N^{-2}\sum_{i=1}^{N}X_{i}V_{i}^{-1}X_{i}\right){\left(N^{-1}\sum_{i=1}^{N}X_{i}^{\prime }V_{i}^{-1}X_{i}+P\right)}^{-1}.$$

Then we can easily construct pointwise standard error for ${\hat{f}}_{p}(t)$, the details of which are omitted here.

### 3.4 Estimation of variance components

We adopt a 4‐step procedure for estimating *C*(*s*,*t*), i.e., **Γ**, and $\sigma_{\epsilon }^{2}$. In step 1, we get an initial estimate ${\hat{\theta }}^{0}$ of ***θ*** by letting $V_{i}=I_{m_{i}}$. In step 2, we obtain residuals $e_{i}=y_{i}-X_{i}{\hat{\theta }}^{0},1\leq i\leq N$, which are then used to estimate *C*(*s*,*t*) and $\sigma_{\epsilon }^{2}$. In step 3, given the estimates $\hat{\Gamma }$ and ${\hat{\sigma }}_{\epsilon }^{2}$ of **Γ** and $\sigma_{\epsilon }^{2}$, respectively, we update $\hat{\theta }$ using (3). Then, in step 4, we again obtain residuals $e_{i}=y_{i}-X_{i}\hat{\theta },1\leq i\leq N$ which are used to produce final estimates of *C*(*s*,*t*) and $\sigma_{\epsilon }^{2}$. While this procedure can be iterated to obtain updated estimates of variance components and fixed effects, in our application, multiple iterations did not result in meaningful changes to estimated quantities.

We use the R package *face* 42 for estimating *C*(*s*,*t*) and $\sigma_{\epsilon }^{2}$ using the residuals **e**_*i*_,1≤*i*≤*N*. The *face* package is based on Xiao et al,^37^ where *C*(*s*,*t*) is decomposed as ∑_1≤*k*,*ℓ*≤*c*_ *B*_*k*_(*s*)*B*_*k*_(*t*)*γ*_*kℓ*_.

### 3.5 Selection of smoothing parameters

Either restricted maximum likelihood or generalized cross validation can be used to select the smoothing parameters *λ*_*k*_(0≤*k*≤*p*) contained in the penalty matrix **P**.^43^ Due to the size of the CONTENT data and the large number of smoothing parameters requiring estimation, we estimate our model using the *bam* function in the *mgcv* package, which uses estimation procedures designed to be much faster for very large datasets.^44^

## 4 DYNAMIC PREDICTION OF LAZ

Consider a new subject with sex covariate *X* and observed growth data $\left\{t_{j},Y_{j},{\tilde{Z}}_{j},1\leq j\leq m\right\}$. For simplicity, we drop the subject index and assume *t*_1_<*t*_2_<⋯<*t*_*m*_. We would like to predict *Y*(*t*) for *t*_*m*_<*t*<*T*_max_ where $T_{\max}=\max(t_{1,m_{1}},t_{2,m_{2}},\text{⋯},t_{i,m_{i}})$. That is, *T*_max_ denotes the time of the latest observed outcome among all subjects on which the model is fit. Extrapolating beyond all observed data is clearly infeasible in the absence of additional information or assumptions on the features of trajectories beyond the observed domain.

From model (1), the model for the new subject is

$$Y_{j}=f_{0}(t_{j})+X\;f_{1}(t_{j})+Z_{j}\;f_{2}(t_{ij})+b(t_{j})+\epsilon_{j},\;1\leq j\leq m.$$

Let $y={\left(Y_{1},\text{⋯},Y_{m}\right)}^{\prime }$, **z**_1_=*X* **1**_*m*_, $z_{2}={\left(Z_{1},\text{⋯},Z_{m}\right)}^{\prime }$, $B_{*}={\left[B(t_{1}),\text{⋯},B(t_{m})\right]}^{\prime }$, and **X**=[**B**_∗_,diag(**z**_1_)**B**_∗_,diag(**z**_2_)**B**_∗_]. Let *b*(*t*)=**B**(*t*)*′* **u**, where $u\in R^{c}$. It can then be derived that the BLUP of **u** is $\tilde{u}=E(u|y)=\Gamma B_{*}^{\prime }{\left(B_{*}\Gamma B_{*}^{\prime }+\sigma_{\epsilon }^{2}I_{m}\right)}^{-1}(y-X\theta )$.^6^ Thus, the BLUP of *b*(*t*) is $B{\left(t\right)}^{\prime }\tilde{u}$, and the BLUP of *Y*(*t*) is $\tilde{Y}(t)=X\theta +B{\left(t\right)}^{\prime }\Gamma B_{*}^{\prime }{\left(B_{*}\Gamma B_{*}^{\prime }+\sigma_{\epsilon }^{2}I_{m}\right)}^{-1}(y-X\theta )$. The variance for $\tilde{Y}(t)-Y(t)$ is $B{\left(t\right)}^{\prime }\left\{\Gamma -\Gamma B_{*}^{\prime }{\left(B_{*}\Gamma B_{*}^{\prime }+\sigma_{\epsilon }^{2}I_{m}\right)}^{-1}B_{*}\Gamma \right\}B(t)+\sigma_{\epsilon }^{2}$, which can be used to construct the approximate 95% pointwise prediction interval for *Y*(*t*) as

$$\tilde{Y}(t)\pm 2\sqrt{B{\left(t\right)}^{\prime }\left\{\Gamma -\Gamma B_{*}^{\prime }{\left(B_{*}\Gamma B_{*}^{\prime }+\sigma_{\epsilon }^{2}I_{m}\right)}^{-1}B_{*}\Gamma \right\}B(t)+\sigma_{\epsilon }^{2}}.$$

In practice, we use the estimated BLUP, where we plug in the estimates of parameters. In particular, we replace *Z*_*i*_=*Z*(*t*_*i*_) by their respective BLUP estimates $\tilde{Z}(t_{i})$.

Note that the width of the prediction intervals will depend on both **Γ** and $\sigma_{\epsilon }^{2}$. Intuitively, the higher the correlation between *Y*(*t*) and observed data, the narrower the prediction intervals will be regardless of the temporal distance *t* and the observed time points. However, in many biological applications, correlation decreases consistently as temporal distance increases. In such scenarios, we expect prediction intervals to generally increase as one attempts to predict further in time from observed time points.

## 5 DATA APPLICATION

**Figure 4:**
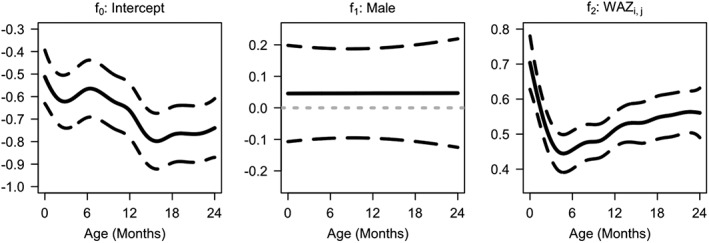
Estimated coefficient functions (solid lines) and associated 95% pointwise confidence intervals (dashed lines). WAZ, weight‐for‐age z‐score

We apply the proposed model (1) to the CONTENT child growth data introduced in Section 2. The estimated coefficient functions are displayed in Figure 4. The baseline/intercept function is negative and generally decreasing. The sex effect is estimated to be near zero at all ages, which is not surprising because the *z*‐scores already account for sex differences. WAZ is positively associated with LAZ with the strongest association near birth and around ages 18 to 24 months.

**Figure 5:**
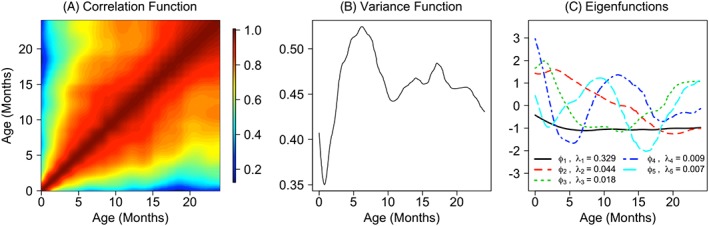
Left to right: A, estimated correlation function; B, estimated variance function; C, first 5 estimated eigenfunctions (ϕ) and corresponding eigenvalues (λ)

Figure 5 displays key features of the estimated covariance function: the correlation function, the variance function Ĉ(t,t),t∈(0,24), and the top 5 estimated eigenfunctions. The within‐curve correlation of the LAZ is relatively high (>0.5 at most pairs of time points), and the variation of LAZ peaks around 6 months. Interestingly, this period corresponds with the time where WAZ has the smallest estimated association with LAZ, suggesting some potential unobserved factor influencing LAZ trajectories during early life. The total variance in the estimated covariance function, which is equal to the sum of the eigenvalues of Ĉ(s,t), is approximately 0.43, and the estimated error variance, ${\hat{\sigma }}_{\epsilon }^{2}$, is 0.033. Accordingly, about 93% of variation in LAZ unexplained by WAZ and sex can be modeled by the random subject‐specific functions.

**Figure 6:**
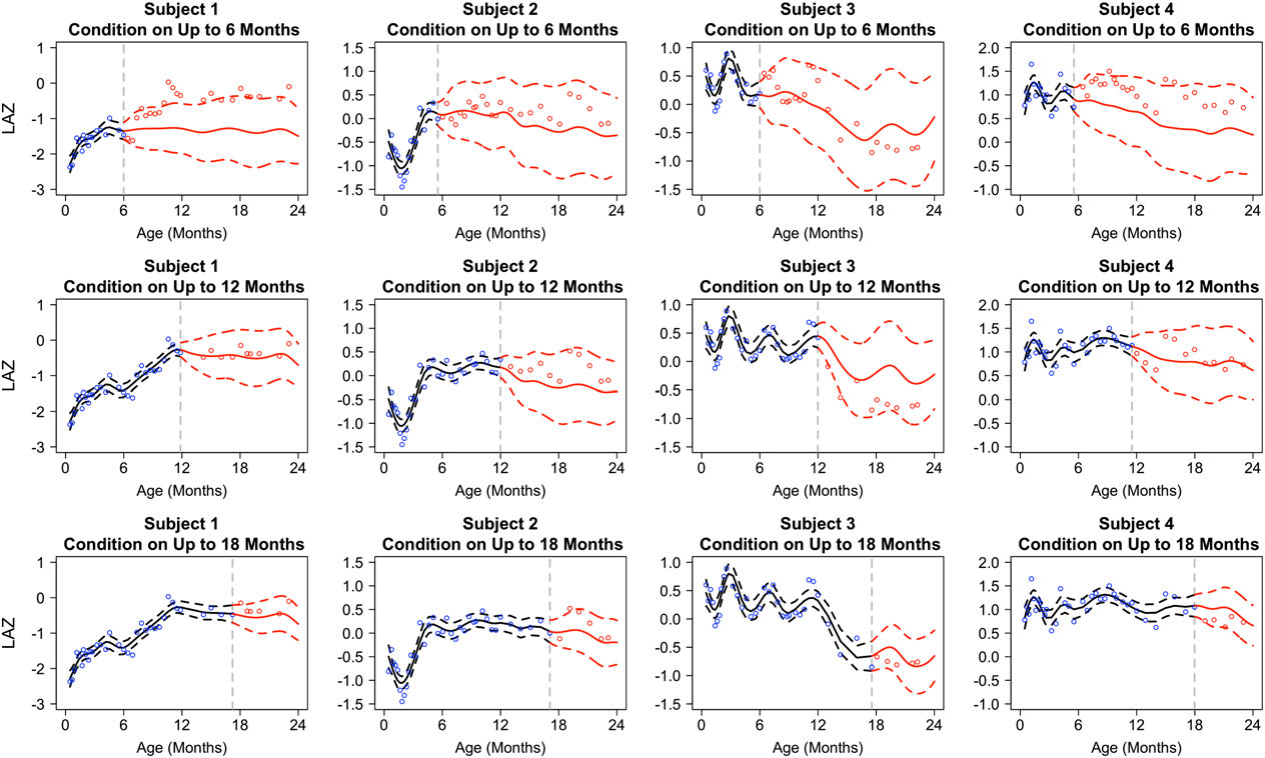
Example of dynamic prediction for 4 subjects. Points represent subjects' observed length‐for‐age z‐score (LAZs), solid black/red lines represent the predicted curves, dashed black/red lines indicate 95% pointwise confidence intervals for the trajectories. Only observed data on and to the left of the vertical grey dashed line (blue points) are used for prediction

To illustrate the idea of dynamic prediction, in Figure 6, we dynamically predict LAZ curves conditioned on up to 6, 12, and 18 months of observed LAZ and WAZ for 4 subjects. This allows for a direct evaluation of future LAZ predictions using partial subject data as a proxy for true out‐of‐sample subject prediction accuracy. Here, each column corresponds to a subject. Only the points on the left of the vertical dashed grey line are used to predict the entire curve. The predicted curves with associated confidence intervals at future time points are shown in red. Unsurprisingly, we see that predictions closer in time to the observed data are generally more accurate. Predictions based on fewer observed data points and farther into the future are generally less accurate and associated with larger uncertainty (wider confidence bands). Moreover, the dynamic prediction intervals generally contain the future actual observed data, indicating the capability of the proposed model to predict future LAZ.

## 6 A SIMULATION STUDY

We conduct a simulation study to investigate the performance of the functional concurrent model in estimating the fixed effects and predicting the individual response curves. For simplicity, hereafter, we denote our proposed model as FCR. We compare our model with an additive model with the same mean structure, but no subject random effects (AM), an additive model with scalar random effects (AMM), and a model with functional random intercept but no covariates (FRI). We find that FCR provides more accurate and efficient estimators of coefficient functions and prediction of subject‐specific curves in terms of both in‐sample and out‐of‐sample dynamic prediction.

### 6.1 Settings

Table 1 presents the list of models we consider. For the AMM, *b*_0*i*_ and *b*_1*i*_ are subject‐specific random intercepts and slopes, respectively. More specifically, (*b*_0*i*_,*b*_1*i*_) are assumed to follow a bivariate normal distribution. For the FCR, *b*_*i*_(·) are subject‐specific random functions. In all models, *ϵ*_*ij*_ are independent and identically distributed random errors with a normal distribution $N(0,\sigma_{\epsilon }^{2})$. We let the generating covariance and coefficient functions be the estimates from applying FCR to the real data. Note that the estimates used in the simulation are slightly different than those presented in Section 5 as we reduced the number of basis functions used to estimate *C*(*s*,*t*), resulting in fewer estimated eigenfunctions and faster computation times for the simulations. This reduced number of basis functions was also used for simulating WAZ as described below.

**Table 1:** Various models

AM:	LAZ_*ij*_=*f*_0_(*t*_*ij*_)+*f*_1_(*t*_*ij*_)Male_*i*_+*f*_2_(*t*_*ij*_)Z_*ij*_+*ϵ*_*ij*_
AMM:	LAZ_*ij*_=*f*_0_(*t*_*ij*_)+*f*_1_(*t*_*ij*_)Male_*i*_+*f*_2_(*t*_*ij*_)Z_*ij*_+*b*_0*i*_+*b*_1*i*_ *t*_*ij*_+*ϵ*_*ij*_
FCR:	LAZ_*ij*_=*f*_0_(*t*_*ij*_)+*f*_1_(*t*_*ij*_)Male_*i*_+*f*_2_(*t*_*ij*_)Z_*ij*_+*b*_*i*_(*t*_*ij*_)+*ϵ*_*ij*_
FRI:	LAZ_*ij*_=*f*_0_(*t*_*ij*_)+*b*_*i*_(*t*_*ij*_)+*ϵ*_*ij*_

Abbreviations: AM, additive model with the same mean structure; AMM, additive model with scalar random effects; FCR, functional concurrent regression; FRI, functional random intercept; LAZ, length‐for‐age *z*‐score.

We compare the FCR to the AM, which ignores within‐subject correlation entirely, the AMM, which allows for subject‐specific random intercepts and slopes for age, and FRI, for which only time is used. We use *mgcv*::gam() for fitting the AM, *mgcv*::gamm() for fitting the AMM, and *face*::face.sparse() for fitting the FRI.

The number of subjects is either *N*=100 or 200, the number of observations *m*_*i*_ is sampled from either a Uniform(15,25) or a Uniform(25,35), and *σ*_*ϵ*_=0.18 or 0.37. In the data, *σ*_*ϵ*_ is estimated to be 0.18 for the FCR. Given *m*_*i*_, the observation times *t*_*ij*_ are sampled uniformly without replacement from an evenly spaced grid of 500 points in the unit interval. The male indicator variable is sampled from a Bernoulli distribution with *p*=0.5.

Both weight‐for‐age *z*‐scores WAZ_*ij*_ and subject‐specific random functional intercepts *b*_*i*_(*t*_*ij*_) are simulated based on the results of applying FRI to the real data. For example, WAZs are generated by ${\text{WAZ}}_{ij}=\mu_{w}(t_{ij})+\sum_{k=1}^{K}\xi_{ik}\phi_{k}(t_{ij})+\epsilon_{ij}^{z}$, where *μ*_*w*_(*t*) is the mean function estimate of WAZ, *ξ*_*ik*_ is generated from *N*(0,*λ*_*k*_), and *λ*_*k*_,*ϕ*_*k*_ are the estimated eigenvalues and eigenfunctions, and $\epsilon_{ij}^{z}∼N(0,\sigma_{z}^{2})$ where *σ*_*z*_=0.16 is the square root of the estimated error variance of WAZ in the CONTENT data. Finally, 500 datasets are generated for each setting. Additional simulation scenarios considering alternative covariance structures and varied signal‐to‐noise ratios yield similar results and hence are included in the Supporting Information.

### 6.2 Results

#### 6.2.1 Estimation of coefficient functions

To evaluate the performance of the FCR for estimating coefficient functions, we use integrated squared error (ISE) defined as $\int_{0}^{1}{\left\{f_{k}(t)-{\hat{f}}_{k}(t)\right\}}^{2}dt$ where *k*=0,1,2. We compare the FCR with the AM and the AMM fits. For each coefficient function, we calculate the medians and interquartile ranges of ISEs of the estimates from each model.

Table 2 indicates that FCR consistently provides more accurate estimates of all coefficient functions than AMM and AM. Table 3 provides the average pointwise coverage probability of estimated coefficient functions across 500 evenly spaced points between 0 and 1. The FCR and AMM perform reasonably well, while the AM performs poorly. The FCR and AMM provide comparable coverage probabilities for estimating the coefficient functions of the time‐invariant covariates (*f*_0_(·) and *f*_1_(·)). However, FCR provides substantially higher coverage probabilities for estimating the coefficient functions of the time‐varying covariate (*f*_2_(·)). All methods compared here seem to give lower than 95% coverage probabilities for *f*_2_. Additional simulations (not shown) indicate that this poor performance can be attributed to the magnitude of the *f*_2_ coefficient and increasing the magnitude of the association between WAZ and the outcome results in close to 95% confidence interval coverage probabilities for FCR.

**Table 2:** 100×Median (interquartile range) of integrated squared error for predicting coefficient functions using FCR, AMM, and AM across 500 simulations for each combination of (N,σ_ϵ_)

		N =100			N =200		
		FCR	AMM	AM	FCR	AMM	AM
*m*_*i*_∼Unif[15,25]							
*σ*_*ϵ*_=0.18	*f*_0_	0.48 (0.71)	0.57 (0.76)	0.63 (0.82)	0.25 (0.40)	0.27 (0.40)	0.34 (0.44)
	*f*_1_	0.74 (1.73)	1.03 (1.83)	1.24 (2.01)	0.38 (0.69)	0.43 (0.82)	0.55 (0.96)
	*f*_2_	0.26 (0.30)	0.30 (0.34)	0.42 (0.53)	0.14 (0.16)	0.15 (0.18)	0.25 (0.24)
*σ*_*ϵ*_=0.37	*f*_0_	0.54 (0.84)	0.57 (0.90)	0.65 (0.95)	0.28 (0.39)	0.28 (0.40)	0.36 (0.43)
	*f*_1_	0.92 (1.68)	1.12 (1.92)	1.29 (1.87)	0.43 (0.84)	0.51 (0.86)	0.66 (1.00)
	*f*_2_	0.30 (0.30)	0.33 (0.38)	0.49 (0.47)	0.16 (0.17)	0.17 (0.17)	0.26 (0.30)
*m*_*i*_∼Unif[25,35]							
*σ*_*ϵ*_=0.18	*f*_0_	0.50 (0.74)	0.60 (0.80)	0.71 (0.91)	0.22 (0.38)	0.26 (0.42)	0.32 (0.44)
	*f*_1_	0.87 (1.46)	1.12 (1.83)	1.31 (1.94)	0.36 (0.73)	0.49 (0.73)	0.57 (0.87)
	*f*_2_	0.21 (0.25)	0.29 (0.37)	0.38 (0.50)	0.11 (0.13)	0.14 (0.18)	0.21 (0.27)
*σ*_*ϵ*_=0.37	*f*_0_	0.54 (0.73)	0.55 (0.84)	0.61 (0.82)	0.27 (0.40)	0.27 (0.42)	0.32 (0.46)
	*f*_1_	0.81 (1.60)	0.92 (1.68)	1.11 (1.73)	0.39 (0.79)	0.50 (0.88)	0.59 (0.93)
	*f*_2_	0.26 (0.26)	0.30 (0.35)	0.44 (0.50)	0.12 (0.13)	0.13 (0.15)	0.23 (0.24)

Abbreviations: AM, additive model with the same mean structure; AMM, additive model with scalar random effects; FCR, functional concurrent regression.

**Table 3:** Average coverage probabilities for 95% confidence bands using FCR, AMM, and AM under various simulation scenarios

		N = 100			N = 200		
		FCR	AMM	AM	FCR	AMM	AM
*m*_*i*_∼Unif[15,25]							
*σ*_*ϵ*_=0.18	*f*_0_	0.93	0.94	0.56	0.93	0.93	0.60
	*f*_1_	0.91	0.93	0.53	0.93	0.94	0.58
	*f*_2_	0.84	0.71	0.63	0.84	0.72	0.64
*σ*_*ϵ*_=0.37	*f*_0_	0.93	0.93	0.60	0.93	0.93	0.63
	*f*_1_	0.93	0.94	0.57	0.94	0.95	0.57
	*f*_2_	0.86	0.79	0.65	0.88	0.80	0.67
*m*_*i*_∼Unif[25,35]							
*σ*_*ϵ*_=0.18	*f*_0_	0.91	0.92	0.50	0.93	0.94	0.55
	*f*_1_	0.92	0.92	0.47	0.94	0.94	0.49
	*f*_2_	0.84	0.64	0.58	0.85	0.65	0.61
*σ*_*ϵ*_=0.37	*f*_0_	0.92	0.94	0.55	0.93	0.94	0.58
	*f*_1_	0.93	0.95	0.53	0.93	0.94	0.51
	*f*_2_	0.86	0.75	0.59	0.90	0.78	0.63

Abbreviations: AM, additive model with the same mean structure; AMM, additive model with scalar random effects; FCR, functional concurrent regression.

Coverage is assessed at 500 equally spaced points between 0 and 24 and then averaged across the 500 points for each simulation scenario.

#### 6.2.2 Dynamic curve prediction

Regarding individual curve predictions, we compare the FCR with the functional principal component model (FRI) and the AMM described in Table 1. We do not include a comparison with the AM because it focuses on the estimation of fixed effects and is therefore not suitable for individual prediction. For each simulated dataset, we calculate the mean ISE (MISE) for predicting all individual curves. The evaluation criteria are the median and interquartile range of the MISEs over all simulated datasets. Mean ISE is calculated using 50 subjects not included during model fitting. Dynamic prediction is performed on 3 nonoverlapping intervals ([8/24,12/24],[14/24,18/24],[20/24,24/24]) using data conditional on up to 3 time points (6/24,12/24,18/24). This results in 6 observed dynamic prediction periods, with MISE calculated for each period separately (see Table 4). For ease of comparison with the CONTENT application, we rescale these time periods to months on the interval 0 to 2 years.

**Table 4:** 10×Median (interquartile range) of mean integrated squared error for dynamically predicting subject‐specific curves using the FCR, fPCA, and AMM across 500 simulations for N=100,m_i_∼Unif(25,35),σ_ϵ_=0.18

		Prediction Time Window			
		8‐12 mo	14‐18 mo	20‐24 mo	
Observed Data	0‐6 mo	2.06 (0.56)	3.41 (0.97)	3.74 (1.20)	FCR
		2.25 (0.64)	3.66 (1.07)	3.88 (1.17)	FRI
		2.21 (0.60)	3.68 (1.07)	4.71 (1.55)	AMM
	0‐12 mo		1.80 (0.49)	2.42 (0.63)	FCR
			1.91 (0.56)	2.54 (0.70)	FRI
			2.17 (0.63)	4.77 (1.47)	AMM
	0‐18 mo			1.18 (0.36)	FCR
				1.27 (0.36)	FRI
				2.50 (0.76)	AMM

Abbreviations: AMM, additive model with scalar random effects; FCR, functional concurrent regression; FRI, functional random intercept; fPCA, functional principal component analysis.

All dynamic prediction errors are assessed using 50 subjects that were not included in the model fitting procedure are used to evaluate the dynamic prediction error presented here. Note that although simulations were performed on the unit interval, the time periods have been rescaled to reflect the time domain of the CONTENT data.

Table 4 reports the results for dynamic prediction error for one simulation scenario (*m*_*i*_∼Unif(25,35),*N*=100,*σ*_*ϵ*_=0.18). Dynamic prediction accuracy increases with the amount and proximity of subject data used in prediction. For example, prediction accuracy for 20 to 24 months using data up to 18 months is better than both: (1) prediction accuracy for 14 to 18 months using data up to 12 months and (2) prediction accuracy for 20 to 24 months using data up to 12 months. Comparing the FCR to the AMM, the improvement in dynamic prediction using up to 12 months of data is substantial, with reductions in MISE between 17% and 53%. However, note that while FCR outperforms FRI, the difference is consistently less than 10%.

Additional simulations (not reported) show that increasing the magnitude of the association between covariates and the outcome increases the relative performance of both FCR and AMM relative to FRI. This makes sense as FRI does not include any covariate information in the model. We therefore recommend using simulation to assess the relative performance of AMM, FRI, and FCR in a particular application under a range of plausible true data generating mechanisms.

## 7 DISCUSSION

We have proposed a dynamic FCR model and provided accompanying code that is easy to use. We applied our method directly to irregularly spaced growth curve data to study the association between length and weight of children and proposed a procedure for performing dynamic prediction. Using the motivating data as a basis for a comprehensive simulation study, our method showed superior performance compared to traditional methods for analyzing growth curve data in terms of estimation and inference on fixed effects as well as both in‐sample and dynamic subject predictions.

As we focus heavily on individual predictions, we would like to underline that simply predicting a new subject for standard mixed effects models is not currently available in the most widely used *R* packages for fitting mixed models (*lme4* and *nlme*), or *refund* for functional mixed effects models. As a result, not only is our method more accurate in terms of subject predictions, it is also easier to make out‐of‐sample predictions from the perspective of an end user. This is particularly relevant for users who want to perform dynamic prediction as the BLUP estimates for the random effects must be recalculated at every point of interest.

In addition to providing a user‐friendly interface, the underlying software has been extensively tested by research groups working on child growth data. While the estimation procedure described in this paper is similar to that proposed in Cederbaum et al,^19^ the procedure has not been implemented in any peer‐reviewed publication. Moreover, our covariance estimation procedure^37^ is fast, accurate, and was designed specifically for sparse functional covariance estimation.

Although our primary focus is on dynamic prediction, the methods are not limited to the specific functional concurrent model considered in the paper. Indeed, it can be used for performing general functional regression with a random functional intercept. This class of models is applicable to a wide variety of study designs and data types. Ultimately, the use of statistical methods depends strongly on the degree to which these methods are accessible. Here, we provide both the method and an estimation procedure that is relatively computationally inexpensive. The accessibility of our code may help facilitate adoption in practice. An *R* package *fcr* has been compiled to fit the proposed dynamic FCR models and will be distributed to CRAN soon.

## Supporting information



Supporting info item

Supporting info item
