# Supplementary material for: Dynamic prediction in functional concurrent regression with an application to child growth
Source: Stat Med. 2017 Dec 11;37(8):1376–88. doi: 10.1002/sim.7582 (PMC5847461; doi:10.1002/sim.7582)
Supplement: Supplementary file 1 — Supporting info item [file SIM-37-1376-s001.pdf]

Supplemental Material for Dynamic Prediction in Functional Concurrent Regression With an Application to Child Growth

Andrew Leroux[1], Luo Xiao[2,3], Ciprian Crainiceanu[1], and William Checkley[1]

[1]*Johns Hopkins University, Baltimore, MD 21205, USA*

[2]*North Carolina State University, Raleigh, NC 27606, USA*

[3]*Email: lxiao5@ncsu.edu*

August 6, 2017

In this document we provide additional simulation results as discussed in the main paper. In total, we examined three generating covariance structures for simulating the random functional intercepts

1. **Data Driven Covariance** $C(s,t) = \sum_{l=1}^{12} \lambda_l \phi_l(s)\phi_l(t)$ where ϕ are eigenfunctions and λ_l are eigenvalues produced from applying FCR to the CONTENT data.

2. **Brownian Motion** $C(s,t) = \sum_{l=1}^{\infty} \lambda_l \phi_l(s)\phi_l(t)$ with eigenvalues $\lambda_l = \frac{1}{(l-1/2)^2\pi^2}$ and eigenfunctions $\phi_l(t) = \sqrt{2}\sin((l-1/2)\pi t)$.

3. **Finite Basis Expansion** $C(s,t) = \sum_{l=1}^{3} \lambda_l \phi_l(s)\phi_l(t)$ where $\lambda_l = 0.5^{l-1}$, $l = 1,2,3$ and $\phi_1(t) = \sqrt{2}\sin(2\pi t)$; $\phi_2(t) = \sqrt{2}\cos(4\pi t)$; and $\phi_3(t) = \sqrt{2}\sin(4\pi t)$.

With the exception of the results for dynamic prediction, Scenario 1 is detailed in the manuscript. Table 3 contains the full dynamic prediction results for this Scenario. Here we see the same general pattern described in the manuscript. That is, the FCR consistently outperforms both FRI and AMM. Uniformly across models, prediction accuracy improves with increased number of subjects, smaller σ_ϵ, and, with the exception of the AMM, prediction error improves with increased number of observations

per subject. Finally, dynamic prediction accuracy increases with the amount and proximity of subject data used in prediction.

For Scenarios 2 and 3, we consider several values of white noise (σ_ϵ) determined by a desired signal-to-noise ratio (SNR). More specifically, we fix the magnitude of the fixed effects f_0, f_1, f_2 and let

$$\sigma_\epsilon^2 = \frac{1}{SNR} \int_{s=0}^{1} \int_{t=0}^{1} C(s,t) ds dt$$

for SNR = 1,2,5,10. We consider all combinations of SNR with $N = 100$ or $N = 200$ subjects and $m_i \sim \text{Unif}[25, 35]$.

Note that for 65-80% of simulated data sets, the AMM failed to converge in Scenario 3. In addition, the AMM failed to converge in a small number of simulated datasets for Scenario 2 (4 simulated data sets out of 4,000 total). All results presented in this document are derived from the simulated data sets where convergence was achieved.

Overall, we find that FCR consistently provides superior performance in terms of estimation and inference of fixed effects, as well as dynamic subject predictions. The degree by which FCR outer-performs the competing methods depends on the covariance structure which generates the random functional intercept, the magnitude of the signal in the random functional intercept, and the various other simulation settings (number of subjects and number of observations per subject). In particular, we find that FCR provides similar levels of improvement over competing methods for Scenarios 1 and 2. For Scenario 3, FCR provides dramatically improved performance over the alternative methods considered.

In Tables 1a and 1b we present the MISE for estimating the fixed effects under Scenarios 2 and 3, respectively. We see that FCR consistently outperforms the AMM

and the AM in both Scenarios. The magnitude of the difference is stark for Scenario 3, with up to 90% lower MISE in some instances.

In Tables 2a and 2b we present the average 95% CI coverage for the fixed effects for Scenarios 2 and 3, respectively. Here we see the FCR consistently outperforms the AMM and the AM in terms of the effect associated with the time-varying covariate, f_2, and generally has comparable or superior coverage for f_0 and f_1. However, we see from Table 2b that FCR under performs in terms of coverage rates for f_0 in Scenario 3. This appears to be due to the fact that in a large number of simulations, f_0 is estimated to be essentially linear, resulting in underestimated standard errors.

In Tables 4 and 5 we present the results of dynamic prediction accuracy. The format of the Table mimics the format of the table in the manuscript with the addition of one extra column per prediction time period to display the results for $N = 100$ and $N = 200$ on the same table. We see that FCR consistently has the lowest prediction error for both Scenarios 2 and 3. FCR outperforms both AMM and FRI by a wide margin for SNR of 1 or 2 under Scenario 3.

(a) Scenario 2 (Brownian Motion)

		N = 100			N = 200		
		FCR	AMM	AM	FCR	AMM	AM
	f_0	0.49 (0.89)	0.53 (0.93)	0.64 (0.93)	0.30 (0.56)	0.33 (0.57)	0.37 (0.56)
SNR = 10	f_1	0.89 (1.80)	1.07 (1.96)	1.20 (2.09)	0.48 (1.00)	0.60 (1.18)	0.69 (1.29)
	f_2	0.30 (0.41)	0.35 (0.55)	0.42 (0.58)	0.14 (0.21)	0.18 (0.27)	0.23 (0.32)
	f_0	0.51 (0.95)	0.60 (1.12)	0.68 (1.12)	0.26 (0.45)	0.28 (0.46)	0.33 (0.47)
SNR = 5	f_1	0.99 (2.01)	1.00 (2.28)	1.31 (2.37)	0.44 (0.88)	0.57 (0.97)	0.68 (1.11)
	f_2	0.28 (0.40)	0.33 (0.42)	0.45 (0.66)	0.13 (0.18)	0.19 (0.25)	0.24 (0.30)
	f_0	0.65 (1.27)	0.71 (1.21)	0.81 (1.23)	0.35 (0.55)	0.35 (0.55)	0.44 (0.61)
SNR = 2	f_1	1.00 (2.39)	1.13 (2.53)	1.31 (2.45)	0.50 (1.25)	0.57 (1.26)	0.73 (1.30)
	f_2	0.33 (0.40)	0.35 (0.47)	0.46 (0.56)	0.16 (0.20)	0.19 (0.22)	0.26 (0.29)
	f_0	0.59 (1.00)	0.64 (1.04)	0.76 (1.09)	0.36 (0.49)	0.37 (0.55)	0.42 (0.51)
SNR = 1	f_1	1.17 (2.27)	1.28 (2.39)	1.40 (2.63)	0.55 (1.11)	0.63 (1.12)	0.82 (1.25)
	f_2	0.37 (0.40)	0.38 (0.48)	0.50 (0.65)	0.23 (0.27)	0.25 (0.25)	0.29 (0.34)

(b) Scenario 3 (Finite Basis Expansion)

		N = 100			N = 200		
		FCR	AMM	AM	FCR	AMM	AM
	f_0	0.24 (0.14)	1.96 (3.18)	2.48 (3.27)	0.21 (0.08)	1.08 (1.74)	1.21 (1.65)
SNR = 10	f_1	0.10 (0.19)	3.58 (6.24)	4.45 (5.88)	0.05 (0.09)	1.82 (3.18)	2.26 (3.21)
	f_2	0.16 (0.17)	1.41 (1.73)	1.59 (1.84)	0.07 (0.07)	0.87 (0.83)	0.84 (0.97)
	f_0	0.29 (0.23)	2.15 (3.79)	2.37 (3.53)	0.23 (0.13)	1.28 (1.78)	1.27 (1.84)
SNR = 5	f_1	0.19 (0.35)	4.07 (6.34)	5.00 (7.44)	0.10 (0.19)	2.26 (3.73)	2.55 (3.56)
	f_2	0.25 (0.19)	1.35 (2.14)	1.52 (1.96)	0.15 (0.16)	0.65 (0.87)	0.78 (0.91)
	f_0	0.43 (0.47)	1.44 (2.97)	2.33 (3.58)	0.29 (0.23)	0.92 (1.87)	1.16 (1.73)
SNR = 2	f_1	0.53 (0.89)	3.46 (5.42)	5.06 (7.49)	0.22 (0.34)	2.15 (2.89)	2.28 (3.31)
	f_2	0.34 (0.28)	1.53 (2.36)	1.62 (2.09)	0.27 (0.18)	0.73 (0.92)	0.89 (1.00)
	f_0	0.63 (0.76)	1.85 (3.08)	2.58 (3.50)	0.38 (0.36)	1.18 (1.80)	1.36 (2.08)
SNR = 1	f_1	0.85 (1.43)	3.21 (5.20)	4.75 (6.75)	0.39 (0.65)	1.70 (3.70)	2.63 (4.41)
	f_2	0.46 (0.41)	1.40 (2.14)	1.85 (2.15)	0.35 (0.25)	0.79 (0.86)	0.87 (0.97)

Table 1: 100×Median (IQR) of integrated squared error for predicting coefficient functions using FCR, AMM, and AM across 500 simulations for each combination of (N, SNR)

(a) Scenario 2 (Brownian Motion)

		N = 100			N = 200		
		FCR	AMM	AM	FCR	AMM	AM
	f_0	0.92	0.93	0.56	0.92	0.92	0.57
SNR = 10	f_1	0.93	0.94	0.56	0.93	0.93	0.52
	f_2	0.85	0.66	0.62	0.88	0.67	0.64
	f_0	0.91	0.93	0.55	0.93	0.93	0.60
SNR = 5	f_1	0.94	0.94	0.55	0.94	0.94	0.54
	f_2	0.87	0.73	0.62	0.89	0.72	0.64
	f_0	0.91	0.92	0.56	0.91	0.92	0.61
SNR = 2	f_1	0.92	0.93	0.56	0.93	0.93	0.56
	f_2	0.87	0.79	0.64	0.90	0.81	0.68
	f_0	0.91	0.92	0.63	0.92	0.92	0.64
SNR = 1	f_1	0.92	0.93	0.59	0.95	0.94	0.61
	f_2	0.87	0.84	0.67	0.90	0.84	0.69

(b) Scenario 3 (Finite Basis Expansion)

		N = 100			N = 200		
		FCR	AMM	AM	FCR	AMM	AM
	f_0	0.58	0.79	0.62	0.54	0.77	0.61
SNR = 10	f_1	0.94	0.81	0.65	0.95	0.81	0.64
	f_2	0.79	0.68	0.65	0.91	0.64	0.64
	f_0	0.67	0.81	0.63	0.59	0.79	0.63
SNR = 5	f_1	0.95	0.82	0.64	0.94	0.81	0.64
	f_2	0.75	0.68	0.65	0.80	0.68	0.65
	f_0	0.81	0.87	0.69	0.71	0.81	0.67
SNR = 2	f_1	0.94	0.86	0.68	0.96	0.86	0.69
	f_2	0.82	0.70	0.69	0.76	0.72	0.68
	f_0	0.88	0.87	0.72	0.81	0.83	0.70
SNR = 1	f_1	0.95	0.89	0.73	0.95	0.88	0.73
	f_2	0.87	0.77	0.73	0.81	0.76	0.72

Table 2: Average coverage probabilities for 95% confidence bands using FCR, AMM, and AM under various simulation scenarios. Coverage is assessed at 500 equally spaced points between 0 and 24 and then averaged across the 500 points for each simulation scenario.

			Prediction Time Window						
			8-12 Months		14-18 Months		20-24 Months		
σ_ϵ	m_i	Observed Data	N = 100	N = 200	N = 100	N = 200	N = 100	N = 200	Model
$\sigma_\epsilon = 0.18$	$m_i \sim$ Unif$[15, 25]$	0-6 Months	2.33 (0.72)	2.24 (0.61)	3.64 (1.05)	3.50 (1.06)	3.96 (1.13)	3.72 (1.14)	FCR
			2.48 (0.73)	2.41 (0.68)	3.79 (1.11)	3.74 (1.09)	4.11 (1.15)	3.85 (1.17)	FRI
			2.42 (0.72)	2.39 (0.65)	3.74 (1.12)	3.73 (1.07)	4.29 (1.29)	4.15 (1.20)	AMM
		0-12 Months			1.93 (0.57)	1.84 (0.53)	2.53 (0.70)	2.41 (0.71)	FCR
					2.02 (0.60)	1.98 (0.56)	2.57 (0.76)	2.49 (0.69)	FRI
					2.10 (0.61)	2.06 (0.59)	3.97 (1.15)	3.90 (1.21)	AMM
		0-18 Months					1.28 (0.40)	1.21 (0.34)	FCR
							1.34 (0.42)	1.28 (0.38)	FRI
							2.34 (0.78)	2.33 (0.69)	AMM
$\sigma_\epsilon = 0.18$	$m_i \sim$ Unif$[25, 35]$	0-6 Months	2.06 (0.56)	1.99 (0.62)	3.41 (0.97)	3.34 (0.95)	3.74 (1.20)	3.64 (1.04)	FCR
			2.25 (0.64)	2.21 (0.66)	3.66 (1.07)	3.58 (1.07)	3.88 (1.17)	3.87 (1.18)	FRI
			2.21 (0.60)	2.17 (0.60)	3.68 (1.07)	3.61 (1.00)	4.71 (1.55)	4.57 (1.34)	AMM
		0-12 Months			1.80 (0.49)	1.69 (0.46)	2.42 (0.63)	2.25 (0.63)	FCR
					1.91 (0.56)	1.82 (0.52)	2.54 (0.70)	2.35 (0.66)	FRI
					2.17 (0.63)	2.11 (0.59)	4.77 (1.47)	4.59 (1.45)	AMM
		0-18 Months					1.18 (0.36)	1.06 (0.33)	FCR
							1.27 (0.36)	1.12 (0.34)	FRI
							2.50 (0.76)	2.34 (0.73)	AMM
$\sigma_\epsilon = 0.37$	$m_i \sim$ Unif$[15, 25]$	0-6 Months	2.56 (0.73)	2.49 (0.77)	3.86 (1.10)	3.82 (1.11)	4.01 (1.16)	3.95 (1.19)	FCR
			2.81 (0.75)	2.73 (0.86)	4.12 (1.25)	4.08 (1.19)	4.26 (1.28)	4.22 (1.25)	FRI
			2.67 (0.78)	2.59 (0.83)	3.98 (1.13)	3.88 (1.08)	4.06 (1.22)	4.10 (1.28)	AMM
		0-12 Months			2.12 (0.63)	2.04 (0.59)	2.62 (0.75)	2.52 (0.74)	FCR
					2.26 (0.70)	2.23 (0.64)	2.72 (0.77)	2.70 (0.77)	FRI
					2.10 (0.65)	2.07 (0.61)	3.14 (0.94)	3.14 (1.02)	AMM
		0-18 Months					1.51 (0.47)	1.43 (0.40)	FCR
							1.59 (0.48)	1.54 (0.42)	FRI
							2.10 (0.63)	2.11 (0.67)	AMM
$\sigma_\epsilon = 0.37$	$m_i \sim$ Unif$[25, 35]$	0-6 Months	2.23 (0.64)	2.19 (0.61)	3.55 (1.07)	3.50 (0.98)	3.82 (1.07)	3.79 (1.12)	FCR
			2.49 (0.69)	2.45 (0.73)	3.81 (1.26)	3.74 (1.12)	4.00 (1.25)	4.05 (1.26)	FRI
			2.35 (0.66)	2.31 (0.64)	3.67 (1.12)	3.58 (1.07)	4.02 (1.22)	4.06 (1.25)	AMM
		0-12 Months			1.95 (0.58)	1.85 (0.52)	2.52 (0.70)	2.42 (0.69)	FCR
					2.10 (0.63)	2.03 (0.55)	2.61 (0.75)	2.56 (0.77)	FRI
					2.04 (0.54)	2.00 (0.57)	3.58 (1.02)	3.49 (1.04)	AMM
		0-18 Months					1.36 (0.39)	1.32 (0.36)	FCR
							1.44 (0.45)	1.40 (0.36)	FRI
							2.18 (0.65)	2.24 (0.61)	AMM

Table 3: 10×Median (IQR) of mean integrated squared error for dynamically predicting subject-specific curves using the FCR, FPCA, and AMM across 500 simulations for each scenario. In each scenario, 50 subjects which were not included in the model fitting procedure are used to evaluate the dynamic prediction error presented here.

		Prediction Time Window						
		8-12 Months		14-18 Months		20-24 Months		
	Observed Data	N = 100	N = 200	N = 100	N = 200	N = 100	N = 200	Model
SNR = 10	0-6 Months	3.00 (0.81)	2.77 (0.76)	6.26 (1.77)	5.79 (1.68)	9.23 (2.54)	8.56 (2.56)	FCR
		3.04 (0.83)	2.93 (0.85)	6.35 (1.67)	5.92 (1.72)	9.19 (2.54)	8.62 (2.55)	FRI
		3.05 (0.82)	2.86 (0.80)	6.64 (1.80)	6.17 (1.86)	10.67 (3.10)	10.02 (2.90)	AMM
	0-12 Months			2.73 (0.75)	2.64 (0.73)	5.92 (1.66)	5.62 (1.72)	FCR
				2.82 (0.69)	2.71 (0.73)	5.92 (1.67)	5.65 (1.70)	FRI
				3.58 (1.04)	3.39 (1.00)	9.00 (2.63)	8.61 (2.33)	AMM
	0-18 Months					2.87 (0.81)	2.67 (0.68)	FCR
						2.89 (0.80)	2.70 (0.65)	FRI
						4.05 (1.17)	3.83 (1.08)	AMM
		8-12 Months		14-18 Months		20-24 Months		
	Observed Data	N = 100	N = 200	N = 100	N = 200	N = 100	N = 200	Model
SNR = 5	0-6 Months	3.11 (0.87)	3.05 (0.78)	6.19 (2.03)	6.04 (1.76)	9.38 (2.80)	8.77 (2.58)	FCR
		3.23 (0.91)	3.22 (0.84)	6.37 (2.10)	6.12 (1.89)	9.31 (2.98)	8.79 (2.41)	FRI
		3.13 (0.93)	3.07 (0.83)	6.49 (2.06)	6.30 (1.81)	10.36 (3.22)	9.80 (2.88)	AMM
	0-12 Months			2.96 (0.82)	2.72 (0.77)	5.99 (1.62)	5.60 (1.75)	FCR
				3.00 (0.82)	2.82 (0.84)	5.97 (1.69)	5.61 (1.68)	FRI
				3.53 (1.00)	3.36 (0.93)	8.60 (2.31)	8.16 (2.36)	AMM
	0-18 Months					2.99 (0.81)	2.81 (0.72)	FCR
						3.02 (0.87)	2.83 (0.78)	FRI
						4.04 (1.15)	3.98 (1.01)	AMM
		8-12 Months		14-18 Months		20-24 Months		
	Observed Data	N = 100	N = 200	N = 100	N = 200	N = 100	N = 200	Model
SNR = 2	0-6 Months	3.40 (0.97)	3.34 (0.94)	6.55 (1.65)	6.49 (1.87)	9.53 (2.66)	9.26 (2.84)	FCR
		3.63 (1.04)	3.60 (0.96)	6.69 (1.90)	6.68 (1.93)	9.45 (2.67)	9.35 (2.76)	FRI
		3.42 (0.91)	3.37 (0.94)	6.58 (1.74)	6.51 (1.98)	9.85 (2.77)	9.60 (2.99)	AMM
	0-12 Months			3.24 (0.89)	3.14 (0.85)	6.30 (1.84)	5.99 (1.86)	FCR
				3.26 (0.88)	3.25 (0.93)	6.31 (1.80)	6.10 (1.93)	FRI
				3.50 (0.93)	3.45 (0.97)	7.86 (2.49)	7.78 (2.12)	AMM
	0-18 Months					3.25 (0.93)	3.12 (0.89)	FCR
						3.24 (0.95)	3.14 (0.91)	FRI
						4.09 (1.30)	4.05 (1.12)	AMM
		8-12 Months		14-18 Months		20-24 Months		
	Observed Data	N = 100	N = 200	N = 100	N = 200	N = 100	N = 200	Model
SNR = 1	0-6 Months	3.72 (1.08)	3.63 (1.01)	6.86 (1.81)	6.76 (1.75)	9.75 (2.90)	9.39 (2.80)	FCR
		4.02 (1.23)	3.95 (1.11)	7.10 (2.07)	7.03 (1.78)	9.80 (3.06)	9.48 (2.76)	FRI
		3.67 (1.04)	3.64 (1.05)	6.78 (1.84)	6.74 (1.78)	9.81 (2.85)	9.49 (2.76)	AMM
	0-12 Months			3.61 (1.07)	3.50 (0.89)	6.47 (1.96)	6.32 (1.83)	FCR
				3.68 (1.01)	3.63 (0.95)	6.53 (1.87)	6.41 (1.83)	FRI
				3.75 (1.09)	3.68 (0.91)	7.49 (2.11)	7.41 (2.10)	AMM
	0-18 Months					3.64 (1.01)	3.49 (1.01)	FCR
						3.65 (0.97)	3.55 (1.06)	FRI
						4.29 (1.18)	4.24 (1.24)	AMM

Table 4: Scenario 2 (Brownian Motion): 10×Median (IQR) of mean integrated squared error for dynamically predicting subject-specific curves using the FCR, FRI, and AMM across 500 simulations. All dynamic prediction errors are assessed using 50 subjects which were not included in the model fitting procedure are used to evaluate the dynamic prediction error presented here. Note that although simulations were performed on the unit interval, the time periods have been re-scaled to reflect the time domain of the CONTENT data.

SNR	Observed Data	Model	8-12 Months, N = 100	8-12 Months, N = 200	14-18 Months, N = 100	14-18 Months, N = 200	20-24 Months, N = 100	20-24 Months, N = 200
SNR = 10	0-6 Months	FCR	6.24 (1.84)	6.11 (1.95)	9.95 (3.05)	9.66 (3.09)	2.60 (0.71)	2.53 (0.67)
SNR = 10	0-6 Months	FRI	7.87 (2.32)	7.72 (2.22)	16.90 (4.60)	16.68 (4.67)	6.49 (1.54)	6.43 (1.50)
SNR = 10	0-6 Months	AMM	12.04 (2.33)	12.16 (3.16)	17.38 (4.10)	16.73 (4.28)	10.69 (3.51)	10.85 (2.66)
SNR = 10	0-12 Months	FCR			1.20 (0.36)	1.12 (0.32)	1.28 (0.32)	1.20 (0.30)
SNR = 10	0-12 Months	FRI			11.22 (3.40)	10.96 (3.40)	4.78 (1.26)	4.57 (1.28)
SNR = 10	0-12 Months	AMM			15.48 (3.13)	14.90 (3.69)	10.32 (2.68)	10.62 (3.18)
SNR = 10	0-18 Months	FCR					0.78 (0.20)	0.71 (0.17)
SNR = 10	0-18 Months	FRI					1.71 (0.46)	1.51 (0.39)
SNR = 10	0-18 Months	AMM					14.62 (3.72)	14.48 (4.43)
SNR = 5	0-6 Months	FCR	7.45 (2.07)	7.38 (2.06)	11.82 (3.45)	11.44 (3.32)	3.08 (0.79)	2.97 (0.78)
SNR = 5	0-6 Months	FRI	8.91 (2.48)	8.72 (2.49)	17.90 (4.83)	17.48 (5.20)	6.89 (1.94)	6.80 (1.72)
SNR = 5	0-6 Months	AMM	12.05 (2.67)	12.04 (2.62)	17.99 (4.01)	17.58 (5.67)	11.03 (2.69)	10.74 (2.60)
SNR = 5	0-12 Months	FCR			1.93 (0.55)	1.76 (0.50)	1.69 (0.44)	1.55 (0.39)
SNR = 5	0-12 Months	FRI			12.03 (3.71)	11.76 (3.54)	5.20 (1.46)	5.03 (1.36)
SNR = 5	0-12 Months	AMM			15.75 (3.91)	15.59 (4.57)	10.44 (2.84)	10.56 (2.43)
SNR = 5	0-18 Months	FCR					1.14 (0.29)	1.02 (0.26)
SNR = 5	0-18 Months	FRI					2.33 (0.72)	2.14 (0.64)
SNR = 5	0-18 Months	AMM					14.35 (4.40)	14.44 (3.90)
SNR = 2	0-6 Months	FCR	8.78 (2.30)	8.64 (2.41)	13.80 (4.26)	13.62 (3.70)	4.28 (1.11)	4.16 (1.09)
SNR = 2	0-6 Months	FRI	10.02 (2.56)	9.89 (2.82)	18.78 (5.38)	18.58 (5.67)	8.02 (2.14)	7.98 (2.08)
SNR = 2	0-6 Months	AMM	12.58 (3.16)	12.02 (2.52)	17.59 (5.60)	17.75 (4.52)	10.91 (2.90)	11.29 (3.54)
SNR = 2	0-12 Months	FCR			3.61 (0.98)	3.45 (1.04)	2.76 (0.70)	2.56 (0.58)
SNR = 2	0-12 Months	FRI			13.14 (4.30)	13.14 (3.81)	6.43 (1.92)	6.07 (1.71)
SNR = 2	0-12 Months	AMM			15.90 (5.01)	15.41 (3.63)	10.53 (2.98)	10.69 (2.99)
SNR = 2	0-18 Months	FCR					2.05 (0.50)	1.83 (0.40)
SNR = 2	0-18 Months	FRI					3.84 (1.13)	3.51 (0.93)
SNR = 2	0-18 Months	AMM					14.30 (4.67)	13.98 (5.13)
SNR = 1	0-6 Months	FCR	9.81 (2.65)	9.44 (2.43)	15.39 (4.08)	15.00 (4.26)	5.49 (1.42)	5.31 (1.31)
SNR = 1	0-6 Months	FRI	10.96 (2.98)	10.65 (2.82)	19.66 (5.50)	19.18 (5.29)	8.92 (2.02)	8.70 (2.23)
SNR = 1	0-6 Months	AMM	12.33 (3.00)	12.26 (2.67)	18.34 (4.54)	17.95 (4.66)	11.97 (2.91)	11.73 (2.75)
SNR = 1	0-12 Months	FCR			5.88 (1.63)	5.43 (1.56)	4.08 (1.02)	3.76 (0.92)
SNR = 1	0-12 Months	FRI			14.62 (4.31)	14.13 (4.15)	7.53 (1.81)	7.16 (1.77)
SNR = 1	0-12 Months	AMM			16.23 (3.62)	15.71 (4.00)	10.96 (2.48)	10.82 (2.48)
SNR = 1	0-18 Months	FCR					3.31 (0.87)	2.92 (0.72)
SNR = 1	0-18 Months	FRI					5.34 (1.45)	4.92 (1.21)
SNR = 1	0-18 Months	AMM					13.99 (3.85)	13.84 (3.11)

Prediction Time Window

Table 5: Scenario 3 (Finite Basis Expansion): 10×Median (IQR) of mean integrated squared error for dynamically predicting subject-specific curves using the FCR, FRI, and AMM across 500 simulations. All dynamic prediction errors are assessed using 50 subjects which were not included in the model fitting procedure are used to evaluate the dynamic prediction error presented here. Note that although simulations were performed on the unit interval, the time periods have been re-scaled to reflect the time domain of the CONTENT data.
