# Supplementary material for: Dynamic prediction in functional concurrent regression with an application to child growth
Source: Stat Med. 2017 Dec 11;37(8):1376–88. doi: 10.1002/sim.7582 (PMC5847461; doi:10.1002/sim.7582)
Supplement: Supplementary file 2 — Supporting info item [file SIM-37-1376-s002.gz › fcr/inst/doc/dynamic-prediction.html]

Dynamic prediction in functional concurrent regression with sparse functional covariates


# Dynamic prediction in functional concurrent regression with sparse functional covariates

#### *Leroux A, Xiao L, Crainiceanu C, Checkley W*

#### *2017-11-07*

This vignette is intended to show users how to fit functional concurrent regression models and then perform dynamic prediction using the *fcr* package. The analysis here mirrors the data application in Leroux et. al (2017).

The *fcr* package was created to fit functional concurrent models when the functional response and predictor are irregularly measured. We note that regularly spaced functional concurrent regression models can be fit using this package, but if dynamic prediction is not of interest and the data are regularly spaced,  
the *refund* package likely provides substantial computational gains over the *fcr* package.

# Model

The *fcr()* function is a wrapper for *mgcv::gam()*/*mgcv::bam()* and *face::face.sparse()*. The mean model specification syntax of *fcr()* is that of *mgcv::gam()*. This function fits models of the form

\[
Y\_ij = f\_0(t\_{ij}) + \beta\_1X\_{i} + f\_2(t\_{ij})Z\_{ij} + f\_3(Z\_{ij}) + f\_4(Z\_{ij}, t\_{ij}) + \cdots + b\_i(t\_{ij}) + \epsilon\_{ij}
\] Where \(b\_i(t\_{ij})\) is denotes a random function intercept and \(\epsilon\_{ij}\) are iid normally distributed noise. We assume the functional random intercept and white noise terms are independent. The *fcr()* function does not currently allow non-identity link functions for the response.

# CONTENT data

The *fcr* package contains simulated child growth data. Below, we present a model fitting procedure and show how to perform dynamic prediction, and plot the results.

In this data, we have two irregularly measured growth indicators, height for age (haz) and weight for age (waz), as well as a time-invariant covariate indicating the sex of the child (Male). We allow for time varying coefficients on both the gender covariate, and concurrently measured waz. More precisely, our model is

\[
\text{HAZ}\_{ij} = f\_0(t\_{ij}) + f\_1(t\_{ij})\text{Male}\_i + f\_2(t\_{ij})WAZ\_{ij} + b\_i(t\_{ij}) + \epsilon\_{ij}
\]

In this data, both \(HAZ\) and \(WAZ\) are measured with noise, and so the first step is to smooth the noisy \(WAZ\) covariate. Here, we smooth using functional principal component analysis via the *face* package.

## Fit the model

```
library(fcr)
## split the data into training and test data
data_full <- content
data      <- subset(data_full, include == 1)  ## data used in model fitting
data_test <- subset(data_full, include == 0)  ## out-of-sample data used in dynamic prediction
rm(list="data_full")

k <- 12 # number of interior knots for fpca (results in k + 3 basis functions)

## Specify the functional domain where we want to make predictions.
## We need to add in the times of observation for the test data
tnew <- sort(unique(c(data$argvals, data_test$argvals)))

###########################################
## Step 1: Smooth time-varying covariate ##
###########################################
dat.waz <- data.frame("y" = data$waz, "subj" = data$subj, argvals = data$argvals)
fit.waz <- face.sparse(dat.waz, newdata = dat.waz, knots = k, argvals.new = tnew)
data$wazPred <- fit.waz$y.pred
```

Once we have the smooth functional covariate, we are ready to fit our model.

```
#####################
## Step 2: Fit fcr ##
#####################

K <- 15 # dimenson of smooth for time varying coefficients

## The mean model in order of the formula presented below:
##     E[Y_{ij}] = f_0(t_{ij}) + f_1(t_{ij})Male_i + f_2(t_{ij})\tilde{WAZ}_{ij}
## note that we do not specify any random effects. That is done internally by fcr()
fit <- fcr(formula = Y ~ s(argvals, k=K, bs="ps") + 
                         s(argvals, by=Male, k=K, bs="ps") + 
                         s(argvals, by=wazPred, bs="ps"),
           argvals = "argvals", subj="subj", data=data, use_bam=TRUE, argvals.new=tnew,
           face.args = list(knots=k, pve=0.99))
```

## Visualize the data

Below we plot the data. The panels in the top row correspond to the estimated \(f\_0,f\_1,f\_2\). The bottom three panels correspond features of the estimate covariance function.

```
#####################
## Examine results ##
#####################

## plot coefficients
par(mfrow=c(2,3),las=1)
for(k in 1:3) plot(fit,select=k)
# plot covariance function
plot(fit, plot.covariance=TRUE)
```

The top three panels present the estimated coefficient functions \(f\_0, f\_1, f\_2\) along with pointwise 95% confidence intervals. The figures are by default created using *plot.gam()*. The bottom three panels visualize the features of the covariance function.

## Prediction

### Dynamic prediction in a functional concurrent model

Dynamic prediction in a functional concurrent model is complicated by the fact that we require a value for the functional covariate at the time where we wish to dynamically predict the outcome of interest.

The solution proposed in Leroux et. al (2017) is to use fpca to dynamically predict the covariate values, then use those predictions as our covariate in the functional concurrent model. As a result, for each new set of dynamic predictions, the functional covariate must be re-estimated.

Suppose we wise to make dynamic predictions for a subject for \(t > t\_m\) for some \(t, t\_m < t\_{\text{max}}\) where \(t\_{\text{max}}\) is the maximum value of the functional domain used in model fitting. The procedure can for dynamic prediction can be summarized in the following steps

1. Create a new data frame (**newdata**) with all available data for the subject up to \(t\_m\)
2. Insert new rows of subject data into **newdata** with **NA** values for the functional covariate and response. The functional domain variable in these rows should indicate the \(t\) for which dynamic predictions are desired.
3. Use fpca (or another prediction procedure) to smooth and dynamically predict the functional covariate at observed times and “unobserved” times, respectively. Do not use this subject’s data beyond \(t\_m\) (if available) to make these predictions.
4. If subjects were used in data fitting, re-assign their subject identifiers to a value not used in model fitting.
5. Supply fitted fcr object and **newdata** to *predict.fcr()*

Note that step (4) above is required by the *fcr()* function to distinguish dynamic prediction from “in-sample” prediction. We provide an example of both in-sample and dynamic prediction in the code below.

### Dynamic versus in-sample prediction in *fcr*

The *predict.fcr()* function allows for both in-sample and dynamic prediction. For in-sample prediction, the estimated BLUPs from *mgcv::bam()*/*mgcv::gam()* are used. Only subjects included in model fitting can be used for in-sample prediction.

In contrast, dynamic prediction is performed on subjects not included in model fitting as indicated by the “subj” identifier. Note that dynamic predictions for subjects that were included in model fitting can be retrieved by assigning them a new subject id in the “newdata” argument to *predict.fcr()*.

The difference between dynamic predictions and in-sample predictions manifests in two ways.

First, suppose we observe data on subject \(i\) at times \(1,2,\ldots,10\) and fit the model on this data. If we supply subject \(i\)’s data for times \(1,2,3\) and **NA** values at times \(4,\ldots,10\), in-sample prediction will provide the same predictions as if we had included all times \(1,\ldots,10\) in the new data. In contrast, dynamic prediction for this subject will re-estimate this subjects’ BLUPs using only the available data (\(t=1,2,3\)). Generally, these two predictions will not be equal.

Second, suppose we’re in the scenario mentioned above, but we supply all the subjects’ data to the predict function. The predicted values will be the same (up to a negligible difference that arises from the differing matrix algebra operations used), but the standard errors will differ. This is because the standard errors calculated for in-sample prediction take into account the estimated covariance of the random effects with fixed effects (see *?predict.gam* for more details). Dynamic predictions, in contrast, assume independence between random effects and fixed effects.

We illustrate these differences in the code below.

```
## do in-sample and dynamic prediction for subject 1
data_pred <- subset(data, subj==1)

## make two separate dataframes for dynamic prediction
## and create make new subject id not used in model fitting
data_pred_dyn      <- data_pred_dyn2      <- data_pred
data_pred_dyn$subj <- data_pred_dyn2$subj <- data_pred_dyn$subj + 10000

## make "observed data" beyond t=0.5 NA
data_pred_dyn$Y[data_pred_dyn$argvals >= 0.5]   <- NA
data_pred_dyn$waz[data_pred_dyn$argvals >= 0.5] <- NA

## calculate dynamic waz scores
data_dyn_waz         <- data.frame("y" = data_pred_dyn$waz,
                                   "subj" = data_pred_dyn$subj,
                                   "argvals" = data_pred_dyn$argvals)
data_pred_dyn$wazPred <- predict(fit.waz, newdata=data_dyn_waz)$y.pred


## perform predictions
in_samp <- predict(fit, newdata=data_pred, se.fit=TRUE)
dyn     <- predict(fit, newdata=data_pred_dyn)
dyn2    <- predict(fit, newdata=data_pred_dyn2)
```

### Perform dynamic prediction on the test data set

Below we show how to perform dynamic prediction for all subjects in the test dataset using data up to \(t\_m = 0.5\). We also supplement the dataset with **NA** values on a fine grid over the range of the functional domain to show how the procedure results in smooth fitted curves and pointwise confidence/prediction intervals.

```
## perform dynamic prediction for test subjects
## on the last 1/2 of the functional domain
data_dyn <- data_test
data_dyn$Y[data_dyn$argvals >= 0.5]   <- NA
data_dyn$waz[data_dyn$argvals >= 0.5] <- NA

## remove smoothed waz -- dyanmic predictions calculated shortly
data_dyn$wazPred <- NULL


set.seed(1012341)
ids <- sample(unique(data_dyn$subj), 4, replace = FALSE)


data_plot <- c()
for(i in 1:length(ids)){
        tmp <- subset(data_dyn, subj %in% ids[i])
        ut_tmp <- tnew[!tnew %in% tmp$argvals & tnew > min(tmp$argvals)]
        n_ut <- length(ut_tmp)
        empty_dat <- data.frame("Y" = rep(NA,n_ut),
                                "Ytrue" = rep(NA,n_ut),
                                "X" = rep(NA,n_ut),
                                "waz.true"=rep(NA,n_ut),
                                "waz"=rep(NA,n_ut),
                                "Male" = rep(tmp$Male[1], n_ut),
                                "argvals"=ut_tmp,
                                "subj"=rep(tmp$subj[1],n_ut),
                                "include"=rep(0,n_ut))
        tmp <- rbind(tmp, empty_dat)
        tmp <- tmp[order(tmp$argvals),]
        
        data_plot <- rbind(data_plot, tmp)
        rm(list=c("tmp","ut_tmp","n_ut","empty_dat"))
}

data_dyn <- data_plot
rm(list=c("data_plot"))

## get dynamic waz predictions
data_dyn_waz     <- data.frame("y" = data_dyn$waz, "subj" = data_dyn$subj, "argvals" = data_dyn$argvals)
data_dyn$wazPred <- predict(fit.waz, newdata=data_dyn_waz)$y.pred

preds <- predict(fit, newdata=data_dyn)$dynamic_predictions
```

```
par(mfrow=c(2,2),las=1)
for(i in 1:length(ids)){
        inx <- which(data_dyn$subj==ids[i])
        inx2 <-which(data_test$subj==ids[i])
        
        yl  <- range(c(preds$fitted.values$y.pred[inx] + 
                               rep(c(1,-1),each=length(inx))*1.96*preds$fitted.values$se.fit.p[inx],
                       data_test$Y[inx2]))

        plot(preds$data$argvals[inx], preds$fitted.values$y.pred[inx],type='l',
             main = paste("Subject",i),xlab="Functional Domain", ylab="Response",
             xlim=c(0,1), ylim=yl)
        lines(preds$data$argvals[inx], lty=2,
              preds$fitted.values$y.pred[inx] - 1.96*preds$fitted.values$se.fit[inx],col='blue')
        lines(preds$data$argvals[inx], lty=2,
              preds$fitted.values$y.pred[inx] + 1.96*preds$fitted.values$se.fit[inx],col='blue')
        
        lines(preds$data$argvals[inx],  lty=2,
              preds$fitted.values$y.pred[inx] - 1.96*preds$fitted.values$se.fit.p[inx],col='red')
        lines(preds$data$argvals[inx],  lty=2,
              preds$fitted.values$y.pred[inx] + 1.96*preds$fitted.values$se.fit.p[inx],col='red')
        axis(1,0.5,labels=expression(t[m]))
        
        points(data_test$argvals[inx2],data_test$Y[inx2])
        
        abline(v=0.5,col='grey',lty=2)
        if(i == 4){
                legend("top",c("Observed Data","fcr() Prediction","95% Confidence Interval","95% Prediction Interval"),
                       col=c("black","black","blue","red"),bty="n",pch=c(1,NA,NA,NA), lty = c(NA,1,2,2))
        }
}
```

Dynamic prediction for four subjects. The solid black line represents the predicted curve, dashed blue and red lines indicate 95% confidence and prediction intervals, respectively. Predictions use only the data up to \(t\_m = 0.5\) to make predictions.
