# Supplementary figures and images for: Dynamic prediction in functional concurrent regression with an application to child growth
Source: Stat Med. 2017 Dec 11;37(8):1376–88. doi: 10.1002/sim.7582 (PMC5847461; doi:10.1002/sim.7582)

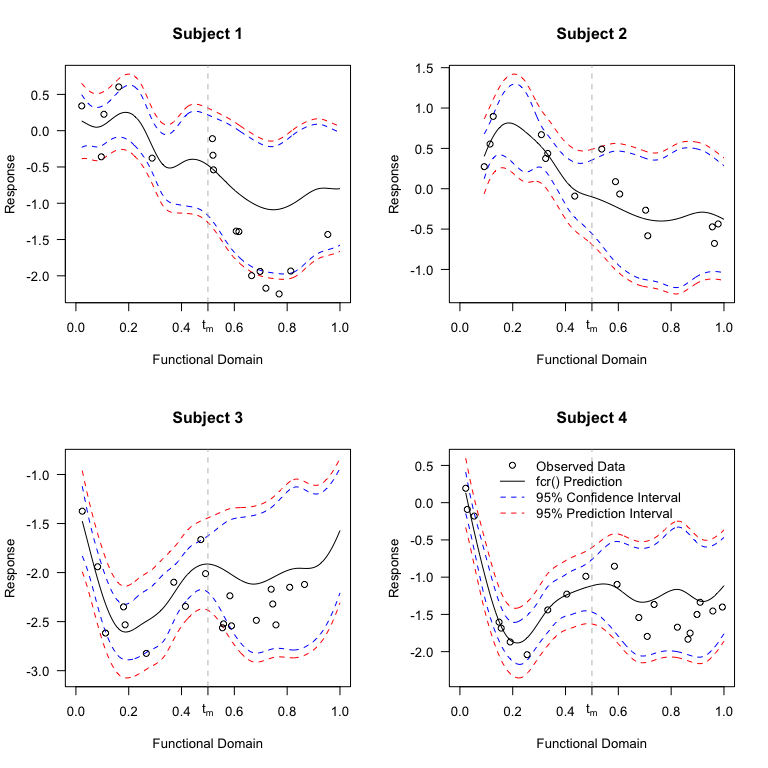

Supplement: Supplementary file 2 — Supporting info item [file SIM-37-1376-s002.gz › fcr/vignettes/plot_test_dyn-1.png]

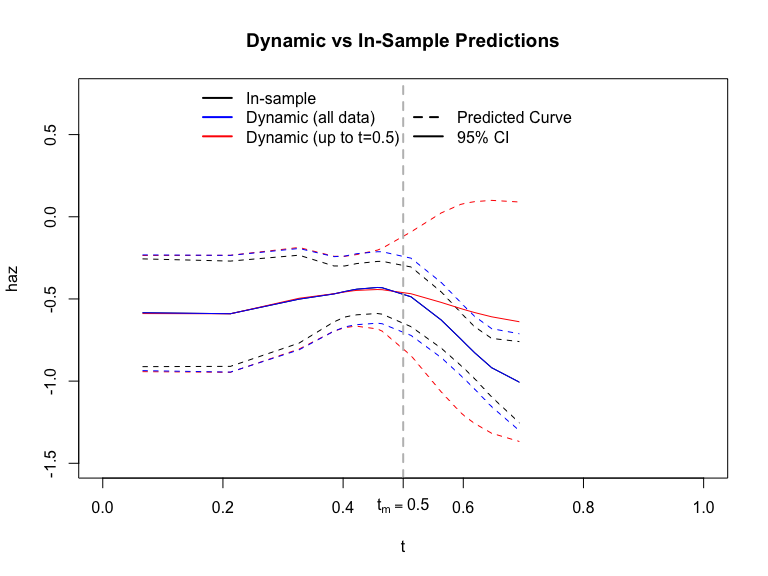

Supplement: Supplementary file 2 — Supporting info item [file SIM-37-1376-s002.gz › fcr/vignettes/prediction_plot-1.png]

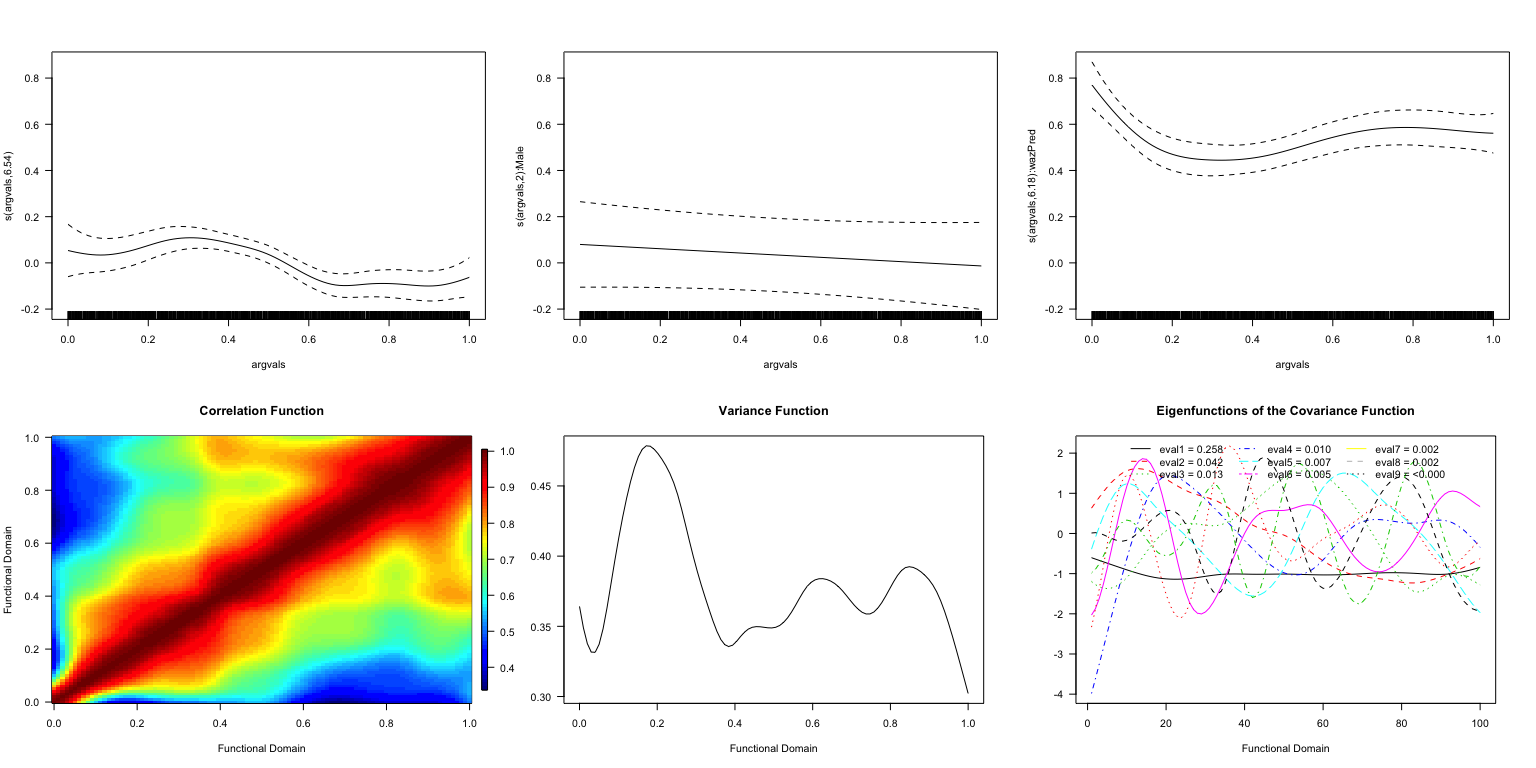

Supplement: Supplementary file 2 — Supporting info item [file SIM-37-1376-s002.gz › fcr/vignettes/visualize-1.png]
